# Dereplication Strategies for Targeted Isolation of New Antitrypanosomal Actinosporins A and B from a Marine Sponge Associated-*Actinokineospora* sp. EG49

**DOI:** 10.3390/md12031220

**Published:** 2014-03-06

**Authors:** Usama Ramadan Abdelmohsen, Cheng Cheng, Christina Viegelmann, Tong Zhang, Tanja Grkovic, Safwat Ahmed, Ronald J. Quinn, Ute Hentschel, RuAngelie Edrada-Ebel

**Affiliations:** 1 Department of Botany II, Julius-von-Sachs Institute for Biological Sciences, University of Würzburg, Julius-von-Sachs-Platz 3, Würzburg D-97082, Germany; E-Mails: usama.ramadan@uni-wuerzburg.de (U.R.A.); cheng.cheng1@uni-wuerzburg.de (C.C.); 2 Strathclyde Institute of Pharmacy and Biomedical Sciences, University of Strathclyde, The John Arbuthnott Building, 161 Cathedral Street, Glasgow G4 0NR, UK; E-Mails: c.cheng@strath.ac.uk (C.C.); christina.viegelmann@strath.ac.uk (C.V.); tong.zhang.101@strath.ac.uk (T.Z.); 3 Eskitis Institute, Griffith University, Brisbane, QLD 4111, Australia; E-Mails: t.grkovic@griffith.edu.au (T.G.); r.quinn@griffith.edu.au (R.J.Q.); 4 Department of Pharmacognosy, Faculty of Pharmacy, Suez Canal University, Ismailia 41522, Egypt; E-Mail: safwat_aa@yahoo.com

**Keywords:** actinosporins, *Spheciospongia vagabunda*, *Actinokineospora*, anti-trypanosomal, dereplication, secondary metabolomics

## Abstract

High resolution Fourier transform mass spectrometry (HRFTMS) and nuclear magnetic resonance (NMR) spectroscopy were employed as complementary metabolomic tools to dereplicate the chemical profile of the new and antitrypanosomally active sponge-associated bacterium *Actinokineospora* sp. EG49 extract. Principal Component (PCA), hierarchical clustering (HCA), and orthogonal partial least square-discriminant analysis (OPLS-DA) were used to evaluate the HRFTMS and NMR data of crude extracts from four different fermentation approaches. Statistical analysis identified the best culture one-strain-many-compounds (OSMAC) condition and extraction procedure, which was used for the isolation of novel bioactive metabolites. As a result, two new *O*-glycosylated angucyclines, named actinosporins A (**1**) and B (**2**), were isolated from the broth culture of *Actinokineospora* sp. strain EG49, which was cultivated from the Red Sea sponge *Spheciospongia vagabunda*. The structures of actinosporins A and B were determined by 1D- and 2D-NMR techniques, as well as high resolution tandem mass spectrometry. Testing for antiparasitic properties showed that actinosporin A exhibited activity against *Trypanosoma brucei brucei* with an *IC*_50_ value of 15 µM; however no activity was detected against *Leishmania major* and *Plasmodium falciparum*, therefore suggesting its selectivity against the parasite *Trypanosoma brucei brucei*; the causative agent of sleeping sickness.

## 1. Introduction

Dereplication refers to the rapid identification of known secondary metabolites and their quantification in crude unfractionated extracts [1,2,3]. This can be a demanding undertaking, because secondary metabolites occur in a wide range of concentrations along with enormous variations in their chemical and physical properties. Therefore, reliable, robust, and selective analytical methods are required to identify secondary metabolites in complex mixtures. To date, LC-UV/MS signatures have been successfully used with AntiBase and MarinLit databases [4]. Owing to the superior sensitivity of mass spectrometry and reproducibility of NMR spectroscopy, they are suitable complementary analytical tools for natural products identification. The large data set produced by NMR and MS experiments requires multivariate analysis for data interpretation, such as principal component analysis (PCA), hierarchical clustering analysis (HCA), and orthogonal partial least square-discriminant analysis (OPLS-DA) [5]. Application of these analytical and statistical methods in metabolomics, facilitates the discovery of potentially novel microbial secondary metabolites as well as the bioassay-guided isolation work [1,2,6,7,8,9,10,11,12]. As an example, Hou and co-workers [13] employed untargeted secondary metabolomics using LC/MS-PCA as an effective strategy for strain prioritization to yield the most chemically diverse and novel natural products for drug discovery. Metabolomics is not yet widely applied as a natural product screening tool, although it has several advantages over the classical bioassay-guided isolation approach. By using combinations of different analytical methods, the bioassay-guided isolation route is shortened and putative identification of potentially new natural products is rapidly achieved [3,14].

Actinomycetes are frequently isolated from different marine, filter-feeding invertebrates, including sponges, corals, tunicates, and jellyfish [15,16,17,18,19]. Marine actinomycetes are still an underexplored source of secondary metabolites and lead molecules with biotechnological, therapeutic, and industrial applications [20,21]. Metabolites from these microorganisms were shown to exhibit diverse activities, such as antibacterial, antifungal, antiparasitic, immunomodulatory, anti-inflammatory, anti-protease, antioxidant, and anticancer activities [22,23,24,25]. These activities are mediated by several classes of natural products, including polyketides, alkaloids, peptides, and terpenes [26,27,28,29]. Members of the genus *Actinokineospora* have been cultivated from terrestrial soils and plants [30,31], and were recently isolated for the first time from a marine sponge [32]. This rare genus is not yet known for the production of secondary metabolites.

Angucyclines are a large family of actinomycete-derived polyketide antibiotics with a four-ring skeleton that constitutes the aglycone part [33,34]. Structurally, angucyclines consist of a benzanthraquinone core, which is usually *C*- or *O*-glycosylated with sugar chains of various lengths, including the three main sugar moieties: olivose, rhodinose, and amicetose, such as rabelomycin and landomycins families (e.g., landomycin P) [35,36]. Angucyclines showed diverse activities, including antitumor, antibacterial, inhibition of platelet aggregation, and enzyme inhibitory activities [37,38]. They were isolated from bacterial genera, such as *Amycolatopsis* and *Nocardia*, and frequently fromthe genus *Streptomyces* [39,40,41], such as most recently from the deep sea sediment-derived *Streptomyces lusitanus* SCSIO LR32 [42].

**Figure 1:**
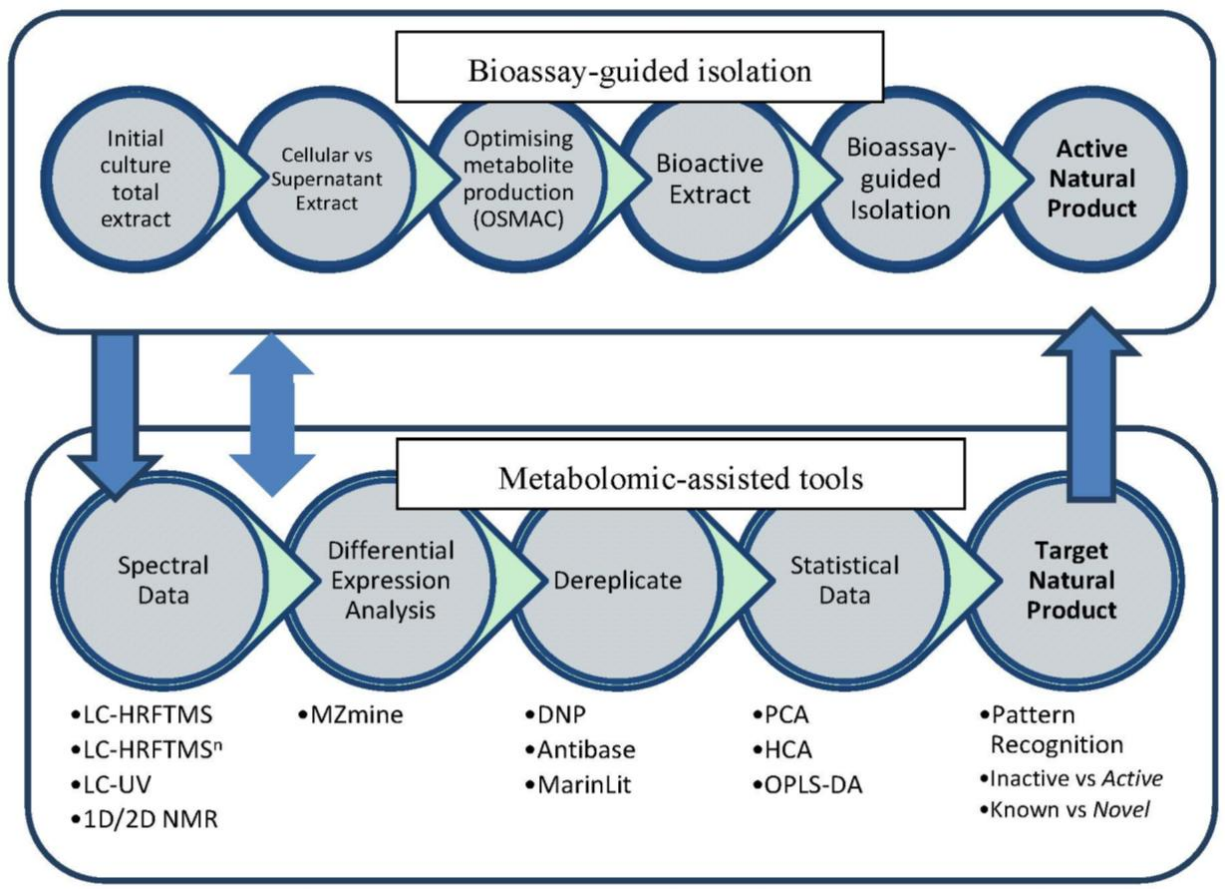
Work flowchart.

The goal of this work was to implement untargeted secondary metabolomics in the dereplication of crude extracts of *Actinokineospora* sp. EG49 [32] that displayed antitrypanosomal activity. Our approach was two-pronged (Figure 1). First, we aimed to optimize the production of the antitrypanosomal metabolites by the “one-strain-many-compounds” (OSMAC) approach [43]. The dereplication strategy employed both MS and NMR to cross validate and quantify the bioactive metabolites in four different fermentation approaches: ISP2 agar, ISP2 liquid broth, ISP2 liquid broth with XAD-16, and ISP2 liquid broth with calcium alginate beads, as well as between two different extraction procedures. Secondly, we aimed to identify putative novel bioactive compounds from the strain EG49.

## 2. Results and Discussion

### 2.1. Metabolomic Profiling of the Crude Ethyl Acetate Extract of ISP2 Agar Culture of EG49

The crude ethyl acetate extract of ISP2 agar culture of *Actinokineospora* sp. EG49 isolate exhibited 100% growth inhibition of *Trypanosoma brucei brucei* S427 at a concentration of 20 µg/mL. This prompted us to perform further chemical work on this bioactive isolate. We used two independent approaches: metabolomics-guided optimisation of the production of the biologically active component and, in parallel, bioassay-guided isolation of the active principle. Dereplication of the secondary metabolites from the antitrypanosomally active isolate *Actinokineospora* sp. strain EG49 was achieved by high resolution Fourier transform mass spectrometry (HRFTMS) using the Exactive-Orbitrap and high resolution NMR. Secondary metabolites were tentatively identified with the aid of existing high resolution MS and NMR records from online and in-house databases, *i.e.*, MarinLit, a database for marine natural products and AntiBase, a database of microbial secondary metabolites. The software tools MZmine 2.10 [44,45] and SIMCA 13.0.2 were utilized to perform differential analysis of sample populations to find significantly expressed features relating to complex biomarkers between parameter variables. Only Antibase and Marinlit databases were employed in this study for a more practical discriminatory search of taxonomically-related microbial metabolites. The limitation of a dereplication study for secondary metabolites, particularly from marine sources, is the difficulty to attain a reference standard for every found “hit” from the database. To ensure the correctness of the identification of the basic structure of the identified peaks, UV, MS/MS data, and NMR spectral data were used to support the results. These combinatory methods of identification were more reliable than utilizing MS/MS data alone for which no global library is available to this date. MS/MS data are highly dependent on various parameters, such as source settings and potential differences between the skimmers as well as between instruments.

**Figure 2:**
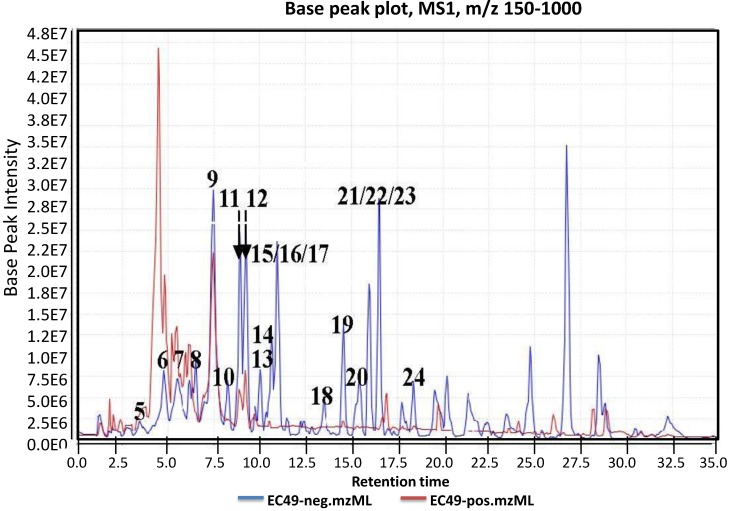
Total ion chromatogram of crude ethyl acetate extract of *Actinokineospora* sp. EG49 ISP2 agar culture in both positive (blue line) and negative (red line) modes. The chromatogram was an output generated from MZmine; a high-throughput differential expression analysis software, which provides algorithms for peak alignment, normalization, and deconvolution. The resulting data sets were then subjected to automated dereplication.

**Figure 3:**
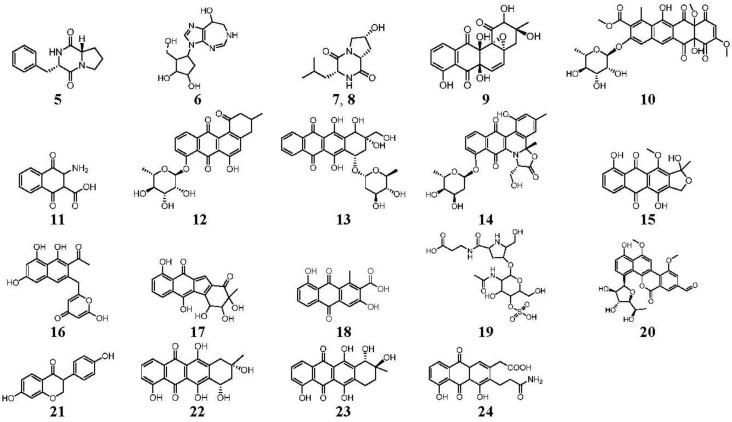
Compounds identified and dereplicated from high resolution mass spectral data sets of the crude ethyl acetate extract of *Actinokineospora* sp. EG49 ISP2 agar culture of by utilizing macros and algorithms that coupled MZmine with both in-house and commercial databases. (Some stereocenters were not assigned due to the presence of other stereoisomers found in the database. As shown in Figure 2, peaks 7 and 8 at different retention times gave the same “hit” compound, which were then numbered accordingly, indicating the possibility of chemical isomers for either peaks. Stereocenters were drawn as found in the database.)

Prior to dereplication, molecular formula prediction was done utilizing MZmine’s algorithm, which employs a combination of empirical techniques that included isotope pattern matching [44]. Using the positive and negative mode electrospray ionization spectral data at a MW tolerance within 5 ppm, known compounds were tentatively identified with AntiBase and MarinLit. Most of the identified compounds were previously isolated from the genus *Streptomyces* (Supplementary Information, Table S1). The identified compounds are highlighted in the total ion chromatogram showing the distribution of the known and unknown compounds (Figure 2). The known compounds detected from the dereplication study are shown in Figure 3. The anthraquinone structure was identified from their typical UV spectrum as well as through the ^13^C NMR spectral data of the crude extract where signals were observed between 160 and 200 ppm (see Supplementary Information, Figure S1). As previously observed by Nielsen’s group [12], the negative mode revealed the presence of a variety of quinone compounds (**9**–**18**, **20**–**24**) (Figure 3). From the unidentified peak list, as shown in Supplementary Information, Table S2, new quinone congeners were detected and the molecular formula prediction, the double bond equivalence, and MS^n^ data are summarized in the table. The MS^2^ and/or MS^3^ data revealed a glycosidic fragment of 146 Da, while MS^3^ fragment gave a Ring Double Bond (RDB) of 14 for the anthraquinone core (see Supplementary Information, Figure S2 for the mass spectral data and Table 1 for a brief illustration of the MS/MS fragmentation for the most intense chromatography peaks). The MS fragmentation experiments confirmed the presence of gylcosidic angucyclines and new compounds against those found in the database. One example is elloramycin E, which is also an anthraquinone congener with just one sugar unit. However, the loss of two 146 Da [C_6_H_10_O_4_] units does suggest that the structure is a glycosidic angucycline congener. 

The presence of anthraquinones was confirmed by UV and NMR spectral data. From the photodiode array (PDA) detector, UV-Vis spectra of individual peaks identified as quinones displayed four transitions above 240 nm with three more intense bands near 250, 270, and 320 nm for a benzenoid and quinonoid moiety, respectively. On the other hand, NMR showed the presence of resonances between 12 and 13 ppm, representing the hydrogen-bound phenolic hydroxyl on the carbonyl group. Quinone analogues have been reported to have antitrypanosomal activity; the inhibitory mechanism of which is assumed to be the induction of oxidative stress against trypanothione reductase, a key enzyme involved in the trypanosomal antioxidant thiol metabolism [46]. Therefore, quinone compounds may contribute to the antitrypanosomal activity of EG49 ISP2 agar extract.

It is clear from the chromatogram that more than half of the peaks were not identified by comparison with the databases, including some of the major components that showed strong peak intensities and good resolution. The *m*/*z* value and the predicted formula of these unidentified compounds are presented in Supplementary Information, Table S2. The high number of unidentified compounds highlights the chemical potential of this strain as a source of new metabolites.

**Table 1:** MS/MS fragmentation of major peaks found in the total ion chromatogram of the crude ethyl acetate extract of *Actinokineospora* sp. EG49 ISP2 agar culture in the negative ionization mode.

RT min	[M − H]^−^	Predicted MF [M − H]^−^	[M − ADDUCT]^−^	MS^2^	MS^3^
8.10	513.1398 14 RDBE	C_26_ H_25_ O_11_ 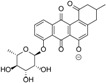 Atramycin A (**12**)	467.13 [M − HCOOH] C_25_ H_23_ O_9_	321.08 [−C_6_ H_10_ O_4_] C_19_ H_13_ O_5_ 13 RDBE 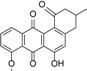	
8.97	657.1822 16 RDBE	C_32_ H_33_ O_15_ 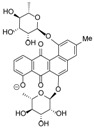 Actinosporin A (**1**)	611.18 [M − HCOOH] C_31_ H_31_ O_13_	465.12 [−C_6_ H_10_ O_4_] C_25_ H_21_ O_9_ 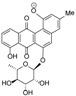	319.06 [−C_6_ H_10_ O_4_] C_19_ H_11_ O_5_ 14 RDBE 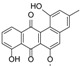
9.85	641.1875 16 RDBE	C_32_ H_33_ O_14_ Unknown	595 [M − HCOOH] C_31_ H_31_ O_12_	451.14 [−C_6_ H_10_ O_4_] C_25_ H_23_ O_8_ 14 RDBE	305.08 [−C_6_ H_10_ O_4_] C_19_ H_13_ O_4_ 13 RDBE
10.60	657.1826 16 RDBE	C_32_ H_33_ O_15_ Elloramycin E * 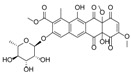 * Elloramycin E not possible due to loss of two sugar units.	611.18 C_31_ H_31_ O_13_ Unknown	465.12 [−C_6_ H_10_ O_4_] C_25_ H_21_ O_9_ 15 RDBE	319.06 [−C_6_ H_10_ O_4_] C_19_ H_11_ O_5_ 14 RDBE
15.37	467.1353 14 RDBE	C_25_ H_23_ O_9_ 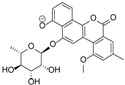 BE-12406-A		320.07 [−C_6_ H_10_ O_4_] C_19_ H_12_ O_5_ 14 RDBE 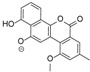	291.07 [−OCH_3_] C_18_ H_11_ O_4_ 13 RDBE 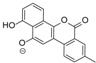
15.98	465.1191 15 RDBE	C_25_ H_21_ O_9_ 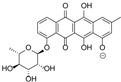 Galtamycinone	449.12 [−O] C_25_ H_21_ O_8_ 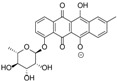	319.06 [−C_6_ H_10_ O_4_] C_19_ H_11_ O_5_ 14 RDBE 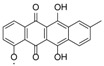	303.07 [−OH] C_19_ H_11_ O_4_ 14 RDB 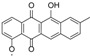

### 2.2. Metabolomic Profiling of Cellular and Supernatant Extracts from Liquid Broth Cultures of EG49

**Figure 4:**
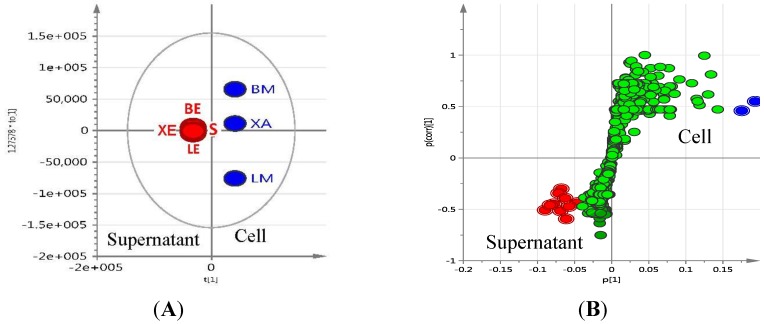
Orthogonal partial least square-d iscriminant analysis (OPLS-DA) (**A**) and S-plot (**B**) of supernatant (red) *vs*. cellular extracts (blue). S (solid); LE (liquid ethyl acetate); LM (liquid methanol); BE (beads ethyl acetate; BM (beads methanol); XE (XAD ethyl acetate); XA (XAD acetone). *R*_2_ = 0.931, *Q*_2_ = 0.995 for OPLS-DA map.

Metabolomic profiling was performed on methanol and acetone cellular extracts of three cell pellets and ethyl acetate extracts of their respective culture supernatants along with an extract obtained from the solid agar medium, as acquired from the cultivation protocol. The MS data were subjected to statistical analysis. PCA was then used to determine the differences of the extracts obtained from various culture conditions and extraction procedures. The degree of similarity between the ethyl acetate supernatant extracts from the four different culture media was higher than those of the methanol and acetone cellular extracts, which exhibited more dispersed and distinct features, as shown from the OPLS-DA map (Figure 4A). An S-plot was primed from the OPLS plot to determine the “end point” compounds, which define unique secondary metabolomes that differentiates the supernatant from the cellular extracts (Figure 4B and Table 2). Different sets of metabolites were extracted by the respective solvents, but with more anthraquinone derivatives being extracted from the supernatant with ethyl acetate than from the cells with acetone and methanol (Table 2).

**Table 2:** The “end point” compounds defined as unique metabolites for every group, as shown on the S-plot in Figure 4B. (P = positive mode; N = negative mode).

Solvent	Ionisation Mode	MS *m/z*	Rt (min)	Chemical Formula	Name
**EtOAc**	N	467.1350	8.27	C_25_H_24_O_9_	Atramycin A (**12**)
**EtOAc**	P	469.1494	8.28	C_25_H_24_O_9_	Atramycin A (**12**)
**EtOAc**	N	513.1404	8.28	C_26_H_25_O_11_	Unknown
**EtOAc**	N	611.1768	8.98	C_31_H_32_O_13_ (new) *	Actinosporin A (**1**)
**EtOAc**	N	657.1828	10.50	C_32_H_34_O_15_	Elloramycin E (**10**)
**EtOAc**	N	501.1407	10.76	C_25_H_26_O_11_ (new) *	Actinosporin B (**2**)
**EtOAc**	N	467.1350	15.13	C_25_H_24_O_9_	BE-12406-A
**EtOAc**	N	449.1247	15.97	C_25_H_22_O_8_	Galtamycinone
**MeOH**	N	377.0860	1.38	C_15_H_9_O_3_N_10_	Unknown
**MeOH**	N	452.2787	21.50	C_25_H_40_O_7_	Unknown

**Figure 5:**
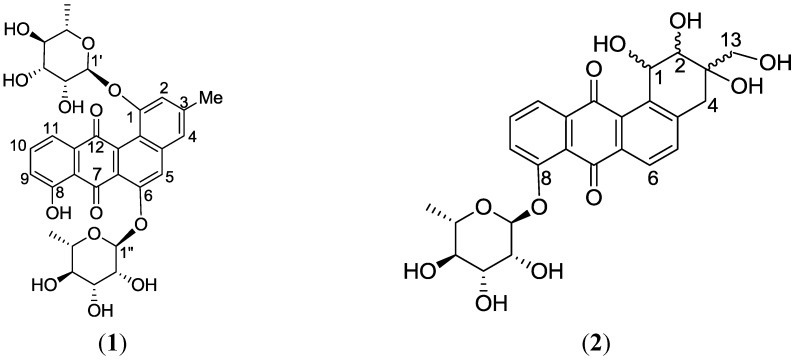
Structures of actinosporins A (**1**) and B (**2**).

* Structures shown in Figure 5.

**Figure 6:**
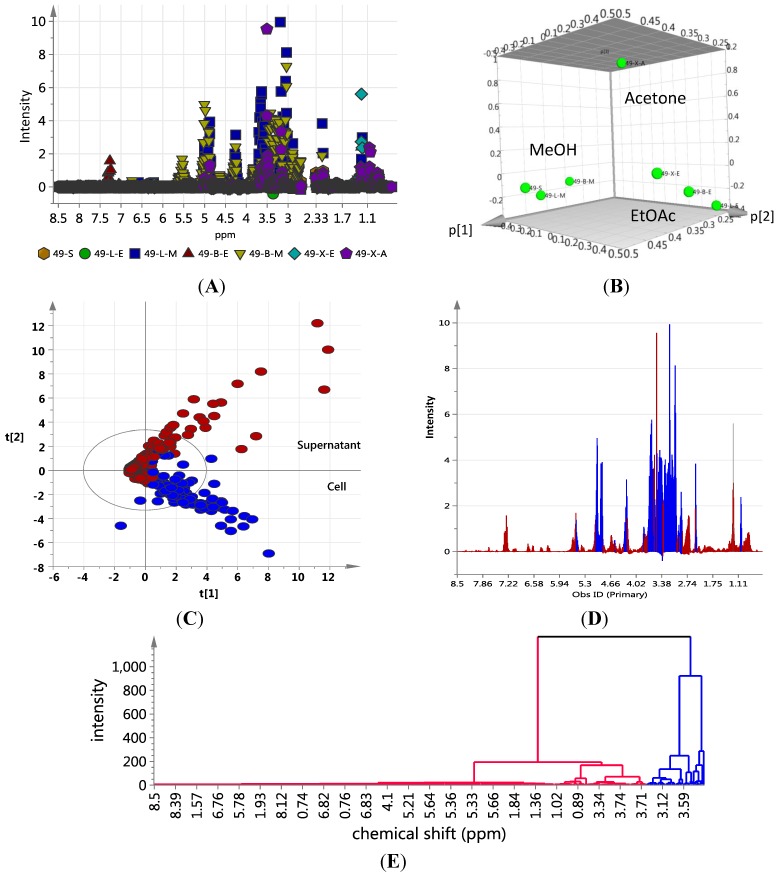
NMR-based dereplication of EG49 extracts: (**A**) Scatter plot of NMR spectral data (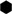
S; ●LE; ■LM; ▲BE; ▼BM; ♦XE; 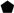
XA); (**B**) 3D Principal component analysis (PCA)-score plot; (**C**) PCA-loading plot of cellular (blue) *vs*. supernatant extracts (red); (**D**) Line plots obtained from the hierarchical clustering analysis of the PCA results as shown in (**E**). The primary observed ID (obs ID) is synonymous to the chemical shift in ppm.

The extraction pattern was validated by subjecting the NMR spectral data (Figure 6A shows the scatter plot of NMR spectral data) to PCA (Figure 6C shows the 3D PCA-score plot and PCA-loading plot, respectively) and line plot (Figure 6D) obtained from the hierarchical clustering analysis (Figure 6E) of the PCA results performed on the NMR spectral data). Figure 6A indicated the presence of anthraquinone compounds to be highly concentrated in ISP2 broth with alginate beads, as shown by the dominance of red triangles (▲ = BE) at *ca.* 7.25 ppm, depicting aromatic proton signals on the anthraquinone moiety. The PCA results gave significant scores of 1.0 and 0.961 for cumulative R_2_X and Q_2_, respectively, which implied a very good prediction model for the variables. The 3D score plot of the different culture extracts showed the cellular XAD-acetone extract as an outlier, whilst the solid agar extract clustered with the methanolic cell extracts (Figure 6B). This could be explained by the different experimental procedures, where the cells were not separated from the agar media prior to extraction in contrast to those of the broth media. Line plots of the hierarchical clustering analysis (HCA) data (Figure 6E) revealed that the methanolic cellular extracts only contained primary polar metabolites (Figure 6D), which were mostly saccharides, as represented by the chemical shifts at *ca.* 5.0 ppm and between 3.0 and 4.0 ppm. The signals from the ethyl acetate extracts showed a more even distribution of resonances between 1.0 and 8.5 ppm in comparison to the methanolic extracts. The presence of signals between 3.0 and 5 ppm signified the presence of sugar moieties in the structure of metabolites found in the supernatant. The distribution of chemical shifts was reflected in the line plot (Figure 6D) obtained from the HCA dendrogram in Figure 6E. The aromatic region was dominated by signals belonging to the ethyl acetate supernatant fractions, which confirmed the presence of glycosidic quinone congeners in conformity with the dereplication study on the mass spectral and mass fragmentation data. The PCA and OPLS-DA of both the NMR and mass spectral data (Figure 3 and Figure 4) consistently showed that the secondary metabolites of interest were secreted into the media. These results highlight the chemical diversity of the supernatants over the cell pellets in *Actinokineospora* sp. EG49 cultures.

### 2.3. Quantification of Actinosporin A in Four Different Culture Media

**Figure 7:**
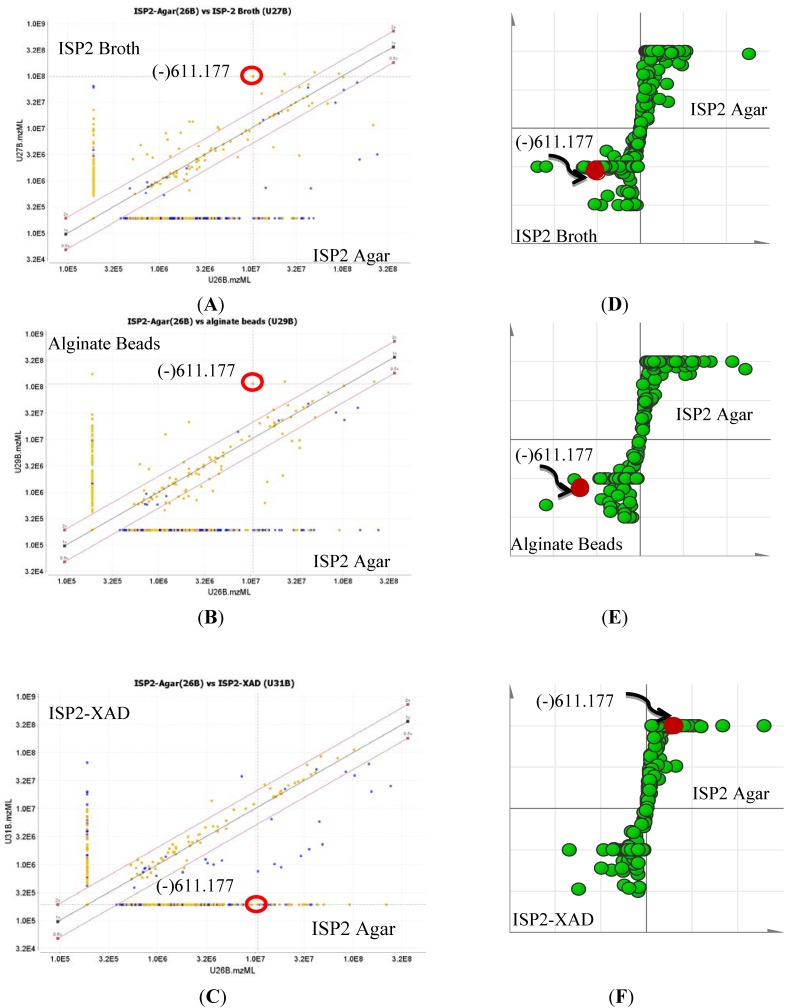
Scatter plot (**A**–**D**, MZmine 2.10) and S-plot (**D**, **E**, SIMCA 13) of the mass spectral data for EG49 ethyl acetate extracts obtained from different culture methods. For the MZmine scatter plots on (**A**–**C**), dots nearer to or on the diagonal lines represent metabolites commonly found in both culture media, while the unique metabolites for each respective media appear above or below the diagonals on the direction of the respective axis representing each of the culture media. For the S-plots on (**D**–**F**), metabolites emerging at the center of the XY axis are the common metabolites for both media, while the unique metabolites for the respective medium are observed at the “end-points” of the sigmoidal curve. Probable quinone congeners eluting between 5 and 30 min are marked yellow on the scatter plots. The ion at *m*/*z* 611.177 with retention time of 8.98 min representing the antitrypanosomal active compound **1** is encircled in red.

**Figure 8:**
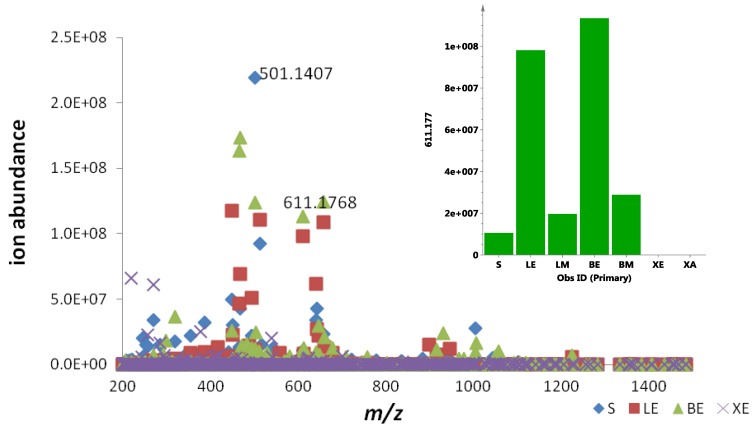
Scatter plots of the dereplicated mass spectral data of EG49 crude extracts obtained from four different growth media: S = ISP2 agar, LE = ISP2 broth, BE = calcium alginate beads, and XE = ISP2 broth with XAD. Inset shows the contribution of actinosporin A in different culture media and extracts.

To optimize production of the biologically active metabolites, dereplication was performed for microbial extracts prepared in triplicates obtained from four different growth media strategies (ISP2 agar, ISP2 broth, ISP2 broth with XAD, and alginate beads), as shown in Figure 7 and Figure 8. Through the scatter- and S-plots (Figure 7), it was shown that unique metabolites were produced in respective culture media. The active antitrypanosomal compound actinosporin A (*m*/*z* 611.1768 [M − H]^−^) with retention time at 8.98 min was shown to be produced at the highest yield in liquid broth (LE) and alginate beads (BE) media (Figure 8), while it was produced in negligible amounts when XAD (XE) was used. There was a significant decrease of probable quinone congeners in the XAD media, which was indicated by the disappearance of dispersed components (represented by yellow dots) in the upper region of the diagonal for the ISP2 XAD extract when comparing the MZmine scatter plots in Figure 7A,B with Figure 7C.

Analyzing the yield of ethyl acetate extract in % *w*/*v*, ISP2 with calcium alginate bead (BE) gave the highest yield of 29.2%, followed by ISP2 agar (S) with 11.9% and ISP2 broth (LE) with 8.9%, while ISP2 with XAD (XE) showed the lowest yield of only 7.3% with extraction efficiencies of 79.9% for BE, 41.8% for LE, and 35.6% for XE. In terms of the production of the antitrypanosomal active metabolite, as shown in Figure 8, the best concentration can be achieved at approximately 1.0E08 ion abundance, using either ISP2 broth (LE) or ISP2 with calcium alginate beads (BE). ISP2 with calcium alginate beads (BE) afforded higher biomass, but the ISP2 broth (LE) offered a more diverse range of interesting congeners, as shown in Figure 7A from the increase in the dispersal of the metabolomes on the *Y* axis for the ISP2 broth extract.

### 2.4. Bioassay-Guided Isolation and Structure Elucidation of the New Actinosporins A and B

Crude extracts of the *Actinokineospora* sp. EG49 strain obtained from four fermentation approaches were tested against *T*. *brucei brucei.* Among all extracts, the ethyl acetate extract from the ISP2 broth culture (LE) was the most active and, therefore, was selected to pursue bioassay-guided isolation of the bioactive metabolites. The antitrypanosomally active ethyl acetate extract was chromatographed on silica gel by gradient elution with CHCl_3_ and MeOH. Fractions exhibiting a growth inhibition of *T*. *brucei brucei* of more than 50% were considered active and subjected to further isolation work (see Figure S3 in Supplementary Information for schematic diagram). The bioactive semi-polar fraction eluting with 60:40 of CHCl_3_:MeOH was further subjected to gel filtration on Sephadex LH-20 by gradient elution with MeOH:H_2_O, commencing with 20% MeOH culminating to 100% MeOH. The LH-20 fraction eluting at 60:40 MeOH:H_2_O exhibited antitrypanosomal activity and final purification was accomplished on reversed-phase HPLC to yield two pure compounds 1 and 2, named actinosporins A and B, respectively (Figure 5). Actinosporin A showed a molecular formula of C_31_H_32_O_13_ by HR-ESIMS (ORBITRAP) analysis (found at *m*/*z* 613.1915 [M + H]^+^), with 16 degrees of unsaturation. HRFT-tandem MS of the molecular ion peak at *m*/*z* 611.1768 [M − H]^−^ for C_31_H_31_O_13_ gave fragment ion peaks at *m*/*z* 465.1177 (C_25_H_21_O_9_, calcd: 465.1186) and 319.0607 (C_19_H_11_O_5_, calcd: 319.0601), and revealed the successive loss of two saccharide units, since the subtracted molecular formula was C_6_H_11_O_4_. This molecular ion peak at *m*/*z* 611.1768 was also found in the above list of unidentified compounds during the dereplication study. The presence of two anomeric protons at δ_H_ 5.48 and 5.66 along with 2 methyl doublets in the aliphatic region indicated the presence of two 6-deoxy rhamnose moieties and further confirmed the characteristic chemical shifts for the anomeric carbons at 99.0 and 99.6 ppm, which suggested the glycosidic nature of the compound. The IR spectrum showed bands at 3389, 3206, 1677 cm^−1^, thus indicating the possible occurrence of carbonyl and hydroxyl functionalities.

The ^1^H-^1^H-COSY spectrum indicated two substructures, along with one methine at δ_H_ 7.69 that appeared as a singlet. The first substructure involved two doublets at δ 7.21 (*J* = 7.3 Hz) and 7.52 (*J* = 7.3 Hz) ppm, and an apparent triplet at δ 7.66 (*J* = 7.3 Hz), which can be attributed to a 1,2,3-trisubstituted aromatic system. The second substructure consisted of two *meta*-coupled aromatic protons at δ_H_ 7.19 and 7.26. A singlet of an aromatic-bound methyl group was observed at δ_H_ 2.47, which coupled to the two *meta*-coupled aromatic resonances. The ^13^C NMR spectrum displayed two quinone carbonyls (δ_C_ 186.2 and 187.5), two anomeric carbons (δ_C_ 99.0 and 99.6), eight oxymethine carbons (δ_C_ 69.6, 69.9, 70:4, 70.6, 70.7, 70.9, 72.4, 72.5), three tertiary carbinols (δ_C_ 152.3, 153.9, 161.2), five shielded sp^2^ methine carbons (δ_C_ 112.2, 116.9, 117.1, 119.6, 122.6), and a distinct signal at δ_C_ 20.8 consistent for an aromatic methyl. Analysis of the COSY, HSQC, and HMBC data (Table 3) showed that the core of the compound had a benz(*α*)anthraquinone backbone, which is characteristic for the angucycline group. ROESY (rotating-frame nuclear Overhauser effect correlation spectroscopy); and HMBC revealed that the two sugars were O-linked to the aglycone at C-1 (^3^*J*_C-H_ correlation between δ_H-1′_ 5.48 with δ_C-1_ 153.9) and the other at C-6 (^3^*J*_C-H_ correlation between H-1′′, δ_H-1_ 5.66 with C-6, δ_C_ 152.3). In addition, ROESY correlations were detected between H-1′ and H-2, as well as between H-1′′ and H-5, confirming the glycosidation at C-1 and C-6, respectively. COSY data deduced the spin systems for the two rhamnose moieties. The two anomeric protons of the two sugars appeared as broad singlets, indicating that the anomeric configuration of the two sugar moieties were α-glycosidically linked. Further confirmation of the equatorial orientation of the anomeric protons on the two rhamnose residues was established via measurement of the one-bond ^13^C–^1^H couplings (^1^*J*_CH_). In a ^13^C-coupled HSQC experiment, the ^1^*J*_CH_ for the two anomeric protons resonating at δ_H_ 5.48 and 5.66 were found to be 172.1 and 172.9 Hz, respectively, consistent with an α-configuration. The relative configuration of the rhamnose residues in **1** was determined by their coupling constants and ROESY data at 600 MHz with *t*_m_ = 400 ms. For sugar A, the large coupling constant for the proton H-4′ (*J* = 9.4 Hz), resulting from diaxial correlations, showed that H-3′, H-4′, and H-5′ had axial orientations. Furthermore, the small coupling constant between H-3′ and H-2′ (*J* = 3.4 Hz) showed that H-2′ was oriented equatorially. Thus, ^1^H NMR coupling constant analysis, COSY and ROESY experiments showed that compound **1** consists of l-rhamnose, which is the naturally occurring isomer among natural products. The relative configuration of sugar B was the same as sugar A, based on the same spectroscopic information. Acid hydrolysis of **1** was performed to yield l-rhamnose, which was confirmed via TLC and comparison to authentic samples. Based on the detailed analysis of the NMR spectra, the structure of compound **1** was determined to be 8-hydroxy-3-methylbenz[*α*]anthracene-7,12-dione-1-*O*-α-l-rhamnopyranose-6-*O*-α-l-rhamnopyranoside, which had not been previously reported. It was designated the trivial name actinosporin A.

**Table 3:** NMR data of actinosporin A (**1**), measured in CD_3_OD-*d*_4_ (^1^H: 600 MHz; ^13^C: 150 MHz).

Position C/H No.	δ_H_, Mult (*J* in Hz)	COSY	δ_C_	Mult	HMBC (δ_H_ to δ_C_)	ROESY
**1**			153.9	C		
**2**	7.19, s	H-3-CH_3_	112.2	CH	C-1, C-3, C-3-CH_3_, C-4, C-12a, C-12b	H-3-CH_3_, H-1A
**3**			142.0	C		
**3-CH_3_**	2.47, s	H-2, H-4	20.8	CH_3_	C-2, C-3, C-4	
**4**	7.26, s	H-3-CH_3_	119.6	CH	C-2, C-3-CH_3_, C-4a, C-12b	H-3-CH_3_, H-5
**4a**			138.9	C		
**5**	7.69, s		116.9	CH	C-4, C-6, C-6a, C-12b	H-4, H-1B, H-5B
**6**			152.3	C		
**6a**			124.0	C		
**7**			186.2	C		
**7a**			115.7	C		
**8**			161.2	C		
**9**	7.21, d (7.3)	H-10, H-11	122.6	CH	C-7a, C-11, C-10	
**10**	7.66, t (7.3)	H-9, H-11	136.1	CH	C-8, C-11, C-11a	
**11**	7.52, d (7.3)	H-9, H-10	117.1	CH	C-7, C-7a, C-9, C-10, C-12	
**11a**			135.5	C		
**12**			187.5	C		
**12a**			140.0	C		
**12b**			116.1	C		
**1′**	5.48, brs	H-2′	99.6	CH	C-1, C-2′, C-5′	H-2
**2′**	4.05, s	H-1′, H-3′	70.4	CH	C-3′, C-4′	
**3′**	3.80, dd (9.4, 3.4)	H-2′, H-4′	70.9	CH	C-4′, C-5′	
**4′**	3.48, t (9.4)	H-3′, H-5′	72.4	CH	C-3′, C-5′, C-6′	H-6′
**5′**	3.76, m	H-6′	69.6	CH	C-6′	H-3′
**6′**	1.25, d (6.3)	H-5′	16.5	CH_3_	C-5′, C-4′	
**1″**	5.66, brs	H-2″	99.0	CH	C-8, C-5″, C-4″	H-5
**2″**	4.22, d (3.4)	H-1″, H-3″	70.6	CH	C-3″, C-4″	
**3″**	4.25, s	H-2″, H-4″	70.7	CH	C-4″	H-5″
**4″**	3.53, t (9.2)	H-3″, H-5″	72.5	CH	C-2″ C-3″, C-6″	
**5″**	3.74, m	H-6″	69.9	CH	C-3″, C-4″, C-6″	
**6″**	1.27, d (6.2)	H-5″	16.6	CH_3_	C-4″, C-5″	

Note: s (singlet); d (doublet); t (triplet); brs (broad singlet).

Actinosporin B was obtained as an orange oil and the mass spectrum revealed the occurrence of *m*/*z* 501.1402 in negative mode and *m*/*z* 503.1554 in positive mode (C_25_H_26_O_11_). This pseudomolecular ion peak was found in the identified compounds list (Table 1) and the structure of **2** was studied by comparing the mass spectral and NMR data with the known compound hit **13**, which was found in AntiBase and proved that the isolated compound was different from compounds reported in the literature. It was of interest to us to elucidate this compound due to its exclusive occurrence in the ISP2 solid agar extract only.

The UV spectrum exhibited a maximum absorption at 254 nm, while a shift of the bands from 310 to 379 nm indicated loss of aromaticity on Ring A. The ^1^H NMR spectrum showed the presence of aromatic protons with peaks from δ_H_ 7.62 to 7.96, which all integrated for one proton each. The COSY spectrum revealed two spin systems in the aromatic region. Correlation between the “pseudo-triplet” at δ_H_ 7.86 and two doublets at δ_H_ 7.96 and 7.71 indicated an ABC spin system. The correlation of two *ortho*-coupled doublets at δ_H_ 7.80 and 7.62 suggested an AB aromatic spin system. The parent structure was determined to be a quinone with one aromatic ABC system (ring D) and another AB system (ring B). The presence of a rhamnose unit was also detected from the COSY spectrum with the characteristic methyl 6.3 Hz doublet at δ_H_ 1.10, which correlated with a proton at δ_H_ 3.53 as well as the broad singlet at δ_H_ 5.65, resulting in a cross peak with a signal at δ_H_ 3.35. Attachment of the rhamnose was deduced to be on C-8 as the proton doublet at C-9 was highly deshielded, as observed in similarly substituted analogues, such as landomycin and atramycin.

Two well-resolved geminal methylene doublets at δ_H_ 3.01 and 2.90 with ^2^*J* of 13.4 Hz were assigned at position 4 due to the benzylic nature of the deshielded chemical shifts as well as their being adjacent to a stereogenic center at C-3. The hydroxyl methylene protons H-13A and H-13B situated on the quaternary chiral carbon C-3A were assigned to the broad doublet signal at δ_H_ 4.93 coupling with its germinal partner proton found underneath the solvent peak at δ_H_ 3.35 as determined by COSY. The hydroxylated broad methine doublet at δ_H_ 5.12 correlated with the broad singlet peak at δ_H_ 4.01 and were assigned to be H-1 and H-2, respectively. For a small yield of less than 1 mg, ^1^H-^1^H-COSY, HSQC/HMQC and HMBC NMR spectral data were collected. However, the HSQC and HMQC did not give any good resolution for further data interpretation. Partial carbon assignments can be deduced from the HMBC data. The methylene doublets at δ_H_ 3.06 and 2.90 for H-4A and H-4B on ring A correlated with carbon signals at δ_C_ 71.7, 72.5, 116.0 133.3, and 140.7 for positions 2, 3, 12b, 12a (^4^*J*_ω_), and 4a respectively, further indicating the loss of unsaturation on ring A. On the other hand, the *ortho* doublet at δ_H_ 7.62 for H-5 on ring B exhibited cross peaks with δ_C_ at 42.7, 116.0, 133.3, and 181.0 (^4^*J*_ω_), for positions 4, 12b, 6a, and 7, respectively. On ring D, cross peaks were observed between the *ortho* doublet at δ_H_ 7.70 for H-9 and δ_C_ at 122.0 for C-11. The *ortho* triplet at δ_H_ 7.87 for H-10 had a correlation with δ_C_ at 135.6 for C-11a and 156.9 for C-8. The 4 ppm upfield shift of C-8 in comparison to compound **1** was additional evidence on the attachment of rhamnose on ring D which was also further confirmed by the correlation of the anomeric proton at δ_H_ 5.65 with δ_C_ 156.9. The methyl doublet at δ_H_ 1.10 had cross peaks with the oxymethine carbons at δ_C_ 71.4 (C-3′) and 71.5 (C-4′), while the anomeric proton correlated with signals at δ_C_ 70.5 (C-2′) and 64.0 (C-5′).

**Figure 9:**
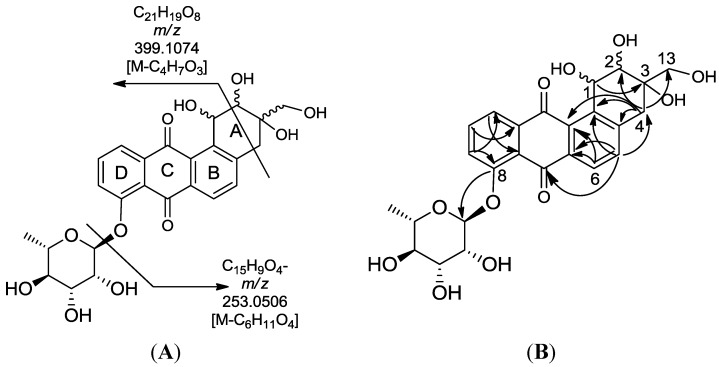
(**A**) Fragmentation of actinosporin B; (**B**) HMBC correlation of actinosporin B. Arrows are from proton (^1^H) to carbon (^13^C).

High resolution FT tandem MS was performed obtaining a fragment ion at *m/z* 399.1079 [M − C_4_H_6_O_3_]^−^ which implied a cross-ring cleavage comparable to a glycan unit [47] and confirmed the presence of a highly hydroxylated saturated ring A. High resolution mass spectral data of the fragment ions verified the presence of four hydroxyl substituents on ring A as shown in Figure 9. Further fragmentation of the ion at *m/z* 399.1079 afforded an ion peak at *m/z* 253.0502 indicating the loss of a saccharide unit as in actinosporin A. The structure was determined as 1,2,3-trihydroxy-3-(hydroxymethyl)-8-rhamnosyl-benz[*α*]anthracene-7,12-dione and assigned the trivial name actinosporin B. 

Actinosporins A and B were tested against the parasites *Leishmania major*, *Trypanosoma brucei brucei,* and *Plasmodium falciparum,* and for cytotoxicity against J774.1 macrophages to verify the original activity of the extract. Antitrypanosomal activity after 48 and 72 h was detected for actinosporin A, which exhibited *IC*_50_ values of 15.47 and 16.44 µM, respectively, with no cytotoxicity against J774.1 macrophages (*IC*_50_ of >200 µM). However, actinosporin B was found to be inactive against *T. brucei*. Human African trypanosomiasis, also known as sleeping sickness, is a fatal disease caused by *T. brucei* affecting mostly the people living in tropical and subtropical regions of the world [46]. The increasing resistance to current therapies and the undesirable side-effects of existing drugs necessitates the development of new, safer, and more potent alternatives to combat this neglected disease. Natural products will continue to play a vital role in the search for new antitrypanosomal drugs [48,49].

## 3. Experimental Section

### 3.1. Fermentation Approaches

#### 3.1.1. Solid Culture

The *Actinokineospora* strain EG49 was fermented for 7 days at 30 °C on 10 ISP2 agar plates. Colonies and agar were cut into small pieces and macerated twice with 500 mL ethyl acetate under continuous shaking at 150 rpm (Edmund Bühler, Hechingen, Germany) overnight. Ethyl acetate marc was filtered (Whatman filter paper, Roth) and the combined filtrates (coded as Solid ethyl acetate) were dried with a rotary evaporator (Heidolph, Schwabach, Germany) under vacuum. The extracts were concentrated under vacuum and stored at 4 °C for chromatographic analysis.

#### 3.1.2. Liquid Culture

One 2 L Erlenmeyer flask containing 1.5 L of ISP2 medium was inoculated with 1 mL of an exponentially growing five-day cell culture of *Actinokineospora* strain EG49 and kept in culture under shaking at 150 rpm and 30 °C for 7 days. After cultivation and filtration, the supernatant was extracted with ethyl acetate (2 × 750 mL). The cells were macerated in 500 mL methanol and shaken for 3 h (150 rpm), and subsequently filtered. The extracts (coded as Liquid ethyl acetate and Liquid methanol, respectively) were concentrated under vacuum and stored at 4 °C prior to further analytical and isolation work.

#### 3.1.3. Liquid Culture with XAD

One 2 L Erlenmeyer flask containing 1.5 L of ISP2 medium supplemented with 20 g/L XAD-16 (Sigma-Aldrich, Dorset, UK) was inoculated with 1 mL of an exponentially growing five-day cell culture of *Actinokineospora* strain EG49 and kept in culture under shaking at 150 rpm at 30 °C for 7 days. After cultivation and filtration, the supernatant was extracted with ethyl acetate (2 × 750 mL). The cells and XAD were shaken with double volumes (2 × 750 mL) of acetone for 3 h, and subsequently filtered. The extracts (coded as XAD ethyl acetate and XAD acetone) were concentrated using a vacuum rotary evaporator and stored at 4 °C prior to analysis. Only cells grown in XAD resin were subject to acetone extraction.

#### 3.1.4. Liquid Culture with Calcium Alginate Beads

An exponentially growing five-day cell culture of *Actinokineospora* strain EG49 (500 mL) was collected by centrifugation at 7155× *g* for 10 min and the pellet was mixed with 4% (*w*/*v*) sodium alginate, containing HEPES buffer (1%). The sodium alginate mixture was slowly dropped with a 5 mL syringe into a 1.5% (*w*/*v*) CaCl_2_ solution at 4 °C under stirring. The produced beads were washed three times with sterile distilled water. Three hundred mL calcium alginate beads with immobilized bacteria were transferred to a 2 L Erlenmeyer flask, containing 1.2 L ISP2 medium and incubated at 30 °C under shaking (150 rpm) for 7 days. After filtration, the beads were extracted by shaking with methanol for 3 h, followed by filtration. The supernatant was extracted with ethyl acetate (2 × 750 mL). The extracts (coded as Beads ethyl acetate and Beads methanol) were concentrated under vacuum and stored at 4 °C prior to further analysis. Blank controls, devoid of bacteria, were used for each approach. The control liquid media were extracted only with ethyl acetate.

### 3.2. General Procedure for Dereplication Study Using MS and NMR Data

Extracts of 1 mg/mL in MeOH were analyzed in triplicates on an Accela HPLC (Thermo Fisher Fisher Scientific, Bremen, Germany), and detected at 280 and 360 nm with a a UV detector and an Exactive-Orbitrap high resolution mass spectrometer (Thermo Fisher Scientific, Bremen, Germany). Media blanks were prepared for comparison reasons. The HPLC column was an ACE (Hichrom Limited, Reading, UK) C18, 75 mm × 3.0 mm, 5 μm column. The mobile phase consisted of purified water (A) and acetonitrile (B) with 0.1% formic acid in each solvent. The gradient program started with 10% B and B was increased linearly in 30 min to 100% B at a flow rate of 300 µL/min and remained isocratic for 5 min before linearly decreasing in 1 min to 10% B. The column was then re-equilibrated with 10% B for 9 min before the next injection. The total analysis time for each sample was 45 min. The injection volume was 10 µL and the tray temperature was maintained at 12 °C. High resolution mass spectrometry was carried out in both positive and negative ESI ionization modes with a spray voltage of 4.5 kV and capillary temperature of 320 °C. The mass range was acquired from *m*/*z* 150 to 1500.

Multi-fragmentation (MS^n^) experiments were accomplished for the negative ionization mode on an Orbitrap analyser, CID (The collision-induced dissociation) was utilized with a normalized collision energy of 35%, activation Q of 0.250 ms, and activation time of 30.000 ms applied on ions of most intense, 2nd most intense, and 3rd most intense peaks for MS^2^, MS^3^, and MS^4^, respectively at an isolation width of 3 microns with 5 microscans. Resolution was at 15,000 m/Δm_50%_, while the minimum ion signal threshold was set to 500. Fragment mass tolerance for molecular formula detection was set to ±5 ppm.

Raw data were initially sliced into two data sets based on the ionization mode, using the MassConvert tool from ProteoWizard [50]. The sliced data sets were imported into MZmine2.10 [51]; a framework for differential analysis of mass spectrometry data. The high-resolution mass spectral (MS^1^) data set was deconvoluted and deisotoped, while mass adducts, fragments, as well as complexes were primarily identified and sorted with MZmine 2.10. The spectra were crop-filtered from 2 to 35 min. The peaks in the samples and blanks were detected using the chromatogram builder. Mass ion peaks were isolated with a centroid detector threshold that was greater than the noise level set to 1.0E4 and an MS level of 1. Following this, the chromatogram builder was used with a minimum time span set to 0.2 min, and the minimum height and *m*/*z* tolerance to 1.0E4 and 0.001 *m*/*z* or 5.0 ppm, respectively. Chromatogram deconvolution was then performed to detect the individual peaks. The local minimum search algorithm (chromatographic threshold: 90%, search minimum in RT range: 0.4 min, minimum relative height: 5%, minimum absolute height: 3.0E4, minimum ratio of peak top/edge: 2, and peak duration range: 0.3–5 min) was applied. Isotopes were also identified using the isotopic peaks grouper (*m*/*z* tolerance: 0.001 *m*/*z* or 5.0 ppm, retention time tolerance: 0.2 absolute (min), maximum charge: 2, and representative isotope: most intense). The retention time normalizer (*m*/*z* tolerance: 0.001 *m*/*z* or 5.0 ppm, retention time tolerance: 5.0 absolute (min), and minimum standard intensity: 5.0E3) was used to reduce inter-batch variation. The peak lists were all aligned using the join aligner parameters set to *m*/*z* tolerance: 0.001 *m*/*z* or 5.0 ppm, weight for *m*/*z*: 20, retention time tolerance: 5.0 relative (%), weight for RT: 20. Missing peaks were detected using the gap filling peak finder (intensity tolerance: 1.0%, *m*/*z* tolerance: 0.001 *m*/*z* or 5.0 ppm, and retention time tolerance of 0.5 absolute (min)). An adduct search was performed for Na-H, K-H, NH_4_, formate, and ACN + H (RT tolerance: 0.2 absolute (min), *m*/*z* tolerance: 0.001 *m*/*z* or 5.0 ppm, max relative adduct peak height: 30%). Additionally, a complex search was performed (ionization method: [M + H]^+^ for ESI positive mode and [M − H]^−^ for ESI negative mode, retention time tolerance: 0.2 absolute (min), *m*/*z* tolerance: 0.001 *m*/*z* or 5.0 ppm, and with maximum complex peak height of 50%). The processed data set was then subjected to molecular formula prediction and peak identification. An accustomed library was created by employing an algorithm to use the molecular formula data set from Antibase^®^ (February 2012) and Marinlit^®^ (September 2012) from which the monoisotopic exact masses were recalculated. The accustomed library was used instead of the manually curated Antibase and MarinLit databases, which do not differentiate between monoisotopic, average, and most abundant mass. The created library was then coupled to MZmine and employed as the custom database for peak identification and dereplication. “Hits” and unidentified peaks (Supplementary Tables S2, and Table 1 and Table 2) were double checked against the MS raw data in Xcalibur 2.1.

For NMR analysis, sample extracts as well as media blanks were prepared at a concentration of 5 mg in 600 μL DMSO-*d_6_*. For the presaturation experiments, the water peak was suppressed and 16 scans were recorded on a 400 MHz NMR system (JEOL, Tokyo, Japan), equipped with the 40TH5AT/FG probe.

Presaturation NMR spectra were processed using MestReNova (Mnova 8.1.0) software (Mestrelab Research, Santiago de Compostela, Spain). The spectral data of samples were stacked, superimposed, and normalized to a constant sum of 100, and each data point was set to be a fraction of the total integral [52]. Baseline correction was accomplished with Whittaker Smoother, apodized with Gaussian 1.0, and smoothing done with Savitzy-Golay. The spectra were then binned, using a spectral width of 0.04 ppm. The NMR and MS spectral data were converted to an ASCII text file and imported into Excel. The data was sorted and organized to remove background peaks that belonged to the culture media. For the NMR data, the solvent peak columns were also deleted. The sorted data was then exported to SIMCA 13.0.2 (Umetrics, Umeå, Sweden) to determine the significant production of unique secondary metabolites between culture variables. Pareto scaling was used for both NMR and MS spectral data sets. PCA, HCA and supervised OPLS-DA were used to evaluate the best culture method.

### 3.3. Purification of Actinosporins A and B

*Actinokineospora* strain EG49 was fermented in 16 2 L-Erlenmeyer flasks, each containing 1 L of ISP2 medium at 30 °C for 7 days under shaking at 150 rpm. After 7 days of growth, the culture broth was filtered and extracted with 10 L ethyl acetate. The ethyl acetate extract was concentrated under reduced pressure and chromatographed on a silica gel column, and eluted with increasing ratios of MeOH in CHCl_3_ from 10% to 100%. The fraction (745 mg) collected with 40% MeOH was further fractionated by gel filtration on Sephadex LH-20 with increasing ratio of MeOH to yield fraction 2 (83 mg) eluted at 60% MeOH in water. Finally, fraction 1 was purified on a semi-preparative HPLC system from Agilent (QuatPump G1311A, Degasser G1322A, Controller ALS G1313A, UV detector G1315A and Fraction collector G1364C). Acetonitrile (MeCN) and water buffered with 0.05% trifluoroacetic acid were used as solvents. Gradient elution with 10% to 100% MeCN in 20 min at a flow rate of 4 mL/min yielded the pure compounds **1** and **2** at a retention time of 7.3 and 9.3 min, respectively.

### 3.4. Compound Characterisation

UV spectra were recorded on a Jasco V650 UV/vis spectrophotometer (Essex, UK), IR spectra were recorded on a Bruker Tensor 27 FT-IR spectrometer (Bremen, Germany). NMR spectra were recorded at 30 °C a Varian 600 MHz Unity INOVA spectrometer (Palo Alto, CA, USA) equipped with a triple resonance cold probe. The ^1^H and ^13^C NMR chemical shifts were referenced to the solvent peaks for CD_3_OD-*d*_4_ at δ_H_ 3.30 and δ_C_ 49.0, respectively. ^13^C NMR spectra were acquired using the Standard Varian s2pul Pulse Sequence. Spectrometer frequency was 150.81 MHz, number of scans used was 5000, receiver gain at 30, relaxation delay set at 0.01 s. Spectral width at 37878.8 Hz. For HMBC experiments, pulse sequence gHMBCAD was used, number of scans was 16, receiver gain at 30, relaxation delay used was 0.8 s, spectral width was 9615.4 Hz for F2 and 33181.3 Hz for F1 with an acquired size of 2048 and 256 k, respectively. The average value for the 2 to 3 bond coupling constant was optimized to 8 Hz. For the HSQC-EDITED experiment, the pulse sequence gHSQC was used. Number of scans was 4, receiver gain at 30, relaxation delay at 1.0000. The average value of the one bond coupling constant was optimized at 140 Hz.

The ROESYAD experiment was utilized to determine the stereochemistry of actinosporin A. However, NOESY experiments were performed for small molecules, at 600 MHz, and exhibited zero or very small NOEs at a range of mixing times. The ROESYAD experiment, on the other hand, did not show any TOCSY artifacts for the correlations in question. The mixing time used for the ROESY experiment was 400 ms.

Actinosporin A (**1**): 3.8 mg of an orange amorphous powder; 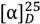 +180 (*c* 0.002, MeOH); UV (MeOH): λ_max_ (log ε) = 227 (4.41), 312 (4.06), 408 (3.61) nm; IR: 3389, 3206, 1677, 1644, 1625, 1364 cm^−1^; ^1^H (CD_3_OD, 600 MHz), and ^13^C NMR (CD_3_OD, 125 MHz) data see Table 3; HR-ESIMS: found at *m*/*z* 613.1915 [M + H]^+^, calcd for C_31_H_33_O_13_, (613.1916), and at *m*/*z* 611.1768 [M − H]^−^, calcd for C_31_H_31_O_13_ (611.1770).

Actinosporin B (**2**): 0.8 mg of an orange amorphous powder; 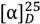 +18.0 (c 0.05, MeOH); UV (MeOH): λ_max_ (log ε) = 254 (4.41), 379 (4.06), 408 (3.61) nm; ^1^H NMR (CD_3_OD, 600 MHz) δ_H_ 7.96 (1H, d, *J*_11,10_ 7.4, H-11), 7.87 (1H, dd, *J*_10,11_ 7.4, *J*_10,9_ 8.2, H-10), 7.79 (1H, d, *J*_5,6_ 7.8, H-5), 7.70 (1H, d, *J*_9,10_ 8.2, H-9), 7.62 (1H, d, *J*_6,5_ 7.8, H-6), 5.64 (1H, br s, H-1′), 4.93 (1H, bs, H-1), 4.83 (1H, d, *J*_3A,3B_ 11.6, H-13A), 4.01 (2H, m, H-2 and H-2′), 3.51 (1H, m, H-5′) 3.33–3.53 (underneath HOD peak, H-13B, H-3′, H-4′), 3.01 (1H, d, *J*_4A,4B_ 13.2, H-4A), 2.90 (d, *J*_4B,4A_ 13.2, H-4B), 1.10 (3H, d, *J*_6′,5′_ 6.3, H-6′); ^13^C NMR (151 MHz, DMSO) δ = 181.0 (C-7), 156.9 (C-8), 140.7 (C-4a), 135.6 (C-11a), 133.27 (C-6a), 133.31 (C-12a), 123.9 (C-7a), 122.0 (C-11), 116.0 (C-12b), 72.5 (C-13), 71.7 (C-3), 71.5 (C-4′), 71.4 (C-3′), 70.5 (C-2′), 70.4 (C-2), 64.0 (C-5′), and 42.7 (C-4); HR-ESIMS: [M − H]^−^ found at *m*/*z* 501.1402, calcd for C_25_H_25_O_11_ (501.1402); [M + H]^+^ found at *m*/*z* 503.1554, calcd for C_25_H_27_O_11_ (503.1548).

### 3.5. Antitrypanosomal Activity Testing

Antitrypanosomal activity was tested following the protocol of Huber and Koella [53]. Briefly, 10^4^ trypanosomes per mL of the *Trypanosoma brucei brucei* strain TC 221 were cultivated in Complete Baltz Medium. Trypanosomes were tested in 96-well plate chambers against different concentrations of test substances at 0.25–40 µM in 1% DMSO to a final volume of 200 µL. For controls, 1% DMSO as well as parasites without any test compound were used simultanously in each plate to show that DMSO did not perturb the results. The plates were then incubated at 37 °C in an atmosphere of 5% CO_2_ for 24 h. After addition of 20 µL of Alamar Blue, the activity was measured after 48 and 72 h by light absorption using an MR 700 Microplate Reader (Dynatech Engineering Ltd., Willenhall, UK) at a wavelength of 550 nm with a reference wavelength of 650 nm The *IC*_50_ values of the test compound were quantified by linear interpolation of three independent measurements.

### 3.6. Cytotoxicity Assay

Macrophages (J774.1) were cultured in complete medium without phenol red in the absence or presence of increasing concentrations of the test compounds (0.25–200 µM) at a cell density of 1 × 10^5^ cells/mL for 24 h, 37 °C, 5% CO_2_, and 95% humidity. Following the addition of 20 μL of Alamar Blue, the plates were incubated and the optical densities were determined after 48 and 72 h in the same manner as described for the anti-trypansomal assay, using a test wavelength of 540 nm and a reference wavelength of 630 nm.

## 4. Conclusions

Metabolomic profiling of *Actinokineospora* sp. EG49 crude extracts utilizing both high resolution mass spectrometry and nuclear magnetic resonance was employed to optimize culture conditions and extraction procedures. We conclude that ISP2 broth culture is more prolific and offers higher chemical diversity than the other three approaches employed in this study. With respect to the isolation of quinone analogues, ethyl acetate extraction of culture supernatants was superior to methanolic extraction of cell pellets. NMR and mass spectral data were successfully used as complementary tools to provide information about the known and the putative new compounds from EG49. Furthermore, actinosporins A and B, new *O*-glycosylated angucyclines, were isolated from the sponge-derived *Actinokineospora* strain EG49. Actinosporin A exhibited activity against *T*. *brucei brucei* with an *IC*_50_ value of 15 µM. This is the first report on the isolation of angucycline derivatives from the genus *Actinokineospora*.

## Supplementary Files

Supplementary File 1 Supplementary Information (PDF, 2321 KB)
