# Supplementary material for: Dereplication Strategies for Targeted Isolation of New Antitrypanosomal Actinosporins A and B from a Marine Sponge Associated-Actinokineospora sp. EG49
Source: Mar Drugs. 2014 Mar 6;12(3):1220–44. doi: 10.3390/md12031220 (PMC3967206; doi:10.3390/md12031220)
Supplement: Supplementary File 1 — Supplementary Information (PDF, 2321 KB) [file marinedrugs-12-01220-s001.pdf]

Supplementary Information

Table S1. Identified compounds of *Actinokineospora* sp. EG49 agar extract with Marinlit database.

Table S2. Unidentified compounds of the crude ethyl acetate extract of ISP2 agar culture of *Actinokineospora* sp. EG49 queried against the Marinlit database (version 2013). Those highlighted ion peaks are probable anthraquinone derivatives.

Figure S1. ^{13}C and Dept Spectra of the crude ethyl acetate extract obtained from the ISP2 agar culture of *Actinokineospora* sp. EG49.

Figure S2. UV, mass spectral data and MS/MS fragmentation data for the most intense chromatographic peaks of the crude ethyl acetate extract obtained from the ISP2 agar culture of *Actinokineospora* sp. EG49.

Figure S3. Bioassay-guided isolation scheme.

Figure S4. Proton spectrum for Actinosporin A.

Figure S5. COSY spectrum for Actinosporin A.

Figure S6. ROESY spectrum for Actinosporin A.

Figure S7. Carbon spectrum for Actinosporin A.

Figure S8. HMBC spectrum for Actinosporin A.

Figure S9. HSQC spectrum for Actinosporin A.

Figure S10. Proton spectrum for Actinosporin B.

Figure S11. COSY spectrum for Actinosporin B (Full Spectrum).

Figure S12. COSY spectrum for Actinosporin B ((aromatic region).

Figure S13. COSY spectrum for Actinosporin B (aliphatic region).

Figure S14. HMBC spectrum for Actinosporin B (aromatic region).

Figure S15. HMBC spectrum for Actinosporin B (aliphatic region).

Table S1. Identified compounds of *Actinokineospora* EG49 extract from the database Marinlit.

	Ionisation Mode	Rt (min)	MS *m/z*	Molecular Weight	Chemical Formula	Name	Tolerance (ppm)	Source	Peak Area
**5**	P	4.03	245.1282196	244.1206	C14H16N2O2	*cis*-cyclo(L-Phe, L-Pro)	1.25	[B] arctic ice bacterium	1.35×10^{7}
**6**	N	5.15	281.1241455	282.1323	C12H18N4O4	8*R*-3[(1*R*,2*S*,3*R*,4*S*)-2,3-Dihydroxy-4-(hydroxymethyl)-cyclopentyl]-3,6,7, 8-tetrahydroimidazo-[4,5-*d*][1,3] diazepin-8-ol	−3	[B] *Saccharothrix*	3.25×10^{7}
**7**	P	5.17	227.1391296	226.1312	C11H18N2O3	cyclo[L-(4-Hydroxyprolinyl)-D-leucine]	2.86	[B] marine bacterium A108	4.43×10^{8}
**8**						*cis*-4-(D)-Hydroxyprolyl diketopiperazine II		[B] marine bacterium A108	
**9**	N	7.72	387.0724182	388.0789	C19H16O9	6a,12a-Dihydroxy-4a,12b-epoxy-(2*R*)-PD-116198	2.07	[B] *Streptomyces phaeochromogenes*	1.55×10^{8}
**10**	N	8.22	657.1825562	658.1892	C32H34O15	Elloramycin E	0.92	[B] *Streptomyces olivaceus*	1.27×10^{8}
**11**	N	8.84	216.03017	217.037	C11H7NO4	2-Amino-3-carboxy-1, 4-naphthoquinone	2.22	[B] *Propionibacterium freudenreichii*	3.41×10^{6}
**12**	P	8.87	453.1546021	452.1466	C25H24O8	Atramycin B	1.65	[B] *Streptomyces atratus*	1.71×10^{7}
**12**	N	8.9	451.1399231	452.1466	C25H24O8	Atramycin B	1.38	[B] *Streptomyces atratus*	7.67×10^{7}
**13**	N	10.03	501.140213	502.147	C25H26O11	F 840020	1.03	0	1.11×10^{8}
**14**	N	10.61	522.140564	523.1473	C27H25NO10	(3aS)-Jadomycin S	1.02	[B] *Streptomyces venezuelae*	1.90×10^{7}
**15**	N	10.96	341.0666809	342.0734	C18H14O7	Fuchurmycin B	1.6	[B] *Streptomyces* sp.	4.19×10^{8}
**16**						PK-8		[B] *Streptomyces roseofulvus* mutant	

Table S1. *Cont.*

	Ionisation Mode	Rt (min)	MS *m/z*	Molecular Weight	Chemical Formula	Name	Tolerance (ppm)	Source	Peak Area
**17**						Momofulvenone-A		[B] *Streptomyces diastatochromogenes*	
**18**	N	13.47	297.0404358	298.0472	C16H10O6	3,8-Dihydroxy-1-methylanthraquinone-2-carboxylic acid; 671-F; DMAC	1.73	[B] *Streptomyces* sp.	8.07×10^{7}
**19**	N	14.57	514.1351318	515.1416	C17H29N3O13S	Bulgecin B	1.63	[B] *Streptomyces* sp.	1.86×10^{7}
**20**	N	15.46	495.1296387	496.1364	C26H24O10	Gilvocarcin A	1.03	[B] *Streptomyces gilvotanareus*	1.39×10^{8}
**21**	N	15.94	253.050705	254.0574	C15H10O4	Daidzein	2.42	[B] *Streptomyces* sp.	4.29×10^{7}
**22**	N	15.94	355.082489	356.0891	C19H16O7	Komodoquinone B	1.98	[B] *Streptomyces* sp.	2.36×10^{8}
**23**						beta1-Rhodomycinone		[B] *Streptomyces purpurascens*	
**24**	P	17.81	370.0922546	369.0843	C19H15NO7	Protetrone	1.8	[B] *Streptomyces aureofaciens*	1.85×10^{7}

Table S2. Unidentified compounds of the crude ethyl acetate extract (S) of ISP2 agar culture of *Actinokineospora* sp. EG49 as obtained from the TIC of both the positive and negative mode of ionization. (*None of the masses detected in the negative mode, corroborated in positive and* vice versa) Molecular formulas were predicted using the MZmine algorithm. Those highlighted ion peaks are probable anthraquinone derivatives determined by MSMS.

Rt (min)	Negative *m/z*	Positive *m/z*	Predicted MF	Tolerance (ppm)
1.25	225.06174		$C_7H_{13}O_8$	0.664
1.93	287.08850		$C_{11}H_{15}N_2O_7$	−0.119
4.03		245.12822	$C_{14}H_{17}O_2N_2$	1.557
4.76	298.09308		$C_{13}H_{16}O_7N$	−1.929
5.17		227.13913	$C_9H_{17}O_2N_5$	1.307
8.22	647.15375		$C_{30}H_{25}O_{12}N_5$	5.178
8.27	243.17139		$C_{12}H_{23}O_3N_2$	0.181
8.27	289.17697		$C_{13}H_{25}N_2O_5$	0.466
8.84	216.03017		$C_{11}H_6O_4N$	−0.282
8.90	633.17438		$C_{21}H_{29}O_{15}N_8$	−1.746
8.90	643.20306		$C_{30}H_{33}O_{13}N_3$	1.545
9.17	357.06175		$C_{18}H_{13}O_8$	0.349
9.74	611.17676		$C_{31}H_{31}O_{13}$	2.076
10.55	370.09338		$C_{19}H_{16}O_7N$	0.932
10.84	687.17505		$C_{26}H_{27}O_{13}N_{10}$	−2.218
10.96	401.08789		$C_{17}H_9O_7N_6$	0.489
11.26	509.10866		$C_{25}H_{15}O_6N_7$	−0.686
11.50	317.11424		$C_{16}H_{17}O_5N_2$	−0.015
12.19	515.11932		$C_{25}H_{23}O_{12}$	−0.465
14.57	497.14540		$C_{26}H_{25}O_{10}$	0.141
14.57	514.13513		$C_{25}H_{24}O_{11}N$	−0.688
14.57	565.13263		$C_{24}H_{25}O_{14}N_2$	2.660
15.29	449.12424		$C_{25}H_{21}O_8$	0.043
16.47	371.07724		$C_{19}H_{15}O_8$	−0.487
16.91		230.24791	$C_{12}H_{30}N_4$	1.497
17.78	737.16619		$C_{30}H_{27}O_{14}N_9$	−8.390
17.78	324.08777		$C_{18}H_{14}O_5N$	−0.203
17.81		370.09225	$C_{16}H_{18}O_{10}$	4.607
18.38	265.14798		$C_{18}H_{19}ON$	3.121
19.54	311.16870		$C_9H_{23}O_6N_6$	0.785
19.54	279.16348		$C_{19}H_{21}NO$	2.534
19.75		258.27921	$C_{16}H_{36}ON$	1.063
21.30	353.20047		$C_{22}H_{27}O3N$	2.259
21.36	325.18439		$C_{10}H_{25}O_6N_6$	0.874
22.29	293.17932		$C_{20}H_{23}NO$	2.651
24.74	257.21234		$C_{15}H_{29}O_3$	0.124
26.75	271.22800		$C_{16}H_{31}O_3$	0.597
28.21		420.33209	$C_{20}H_{44}O_5N_4$	1.026
28.48	285.24356		$C_{17}H_{33}O_3$	0.252
28.84	269.21237		$C_{16}H_{29}O_3$	0.899
28.87		282.27917	$C_{18}H_{36}ON$	1.114
30.49	360.25473		$C_{22}H_{34}NO_3$	1.035

Figure S1. ^{13}C and DEPT spectra of the crude ethyl acetate extract obtained from the ISP2 agar culture of *Actinokineospora* sp. EG49.

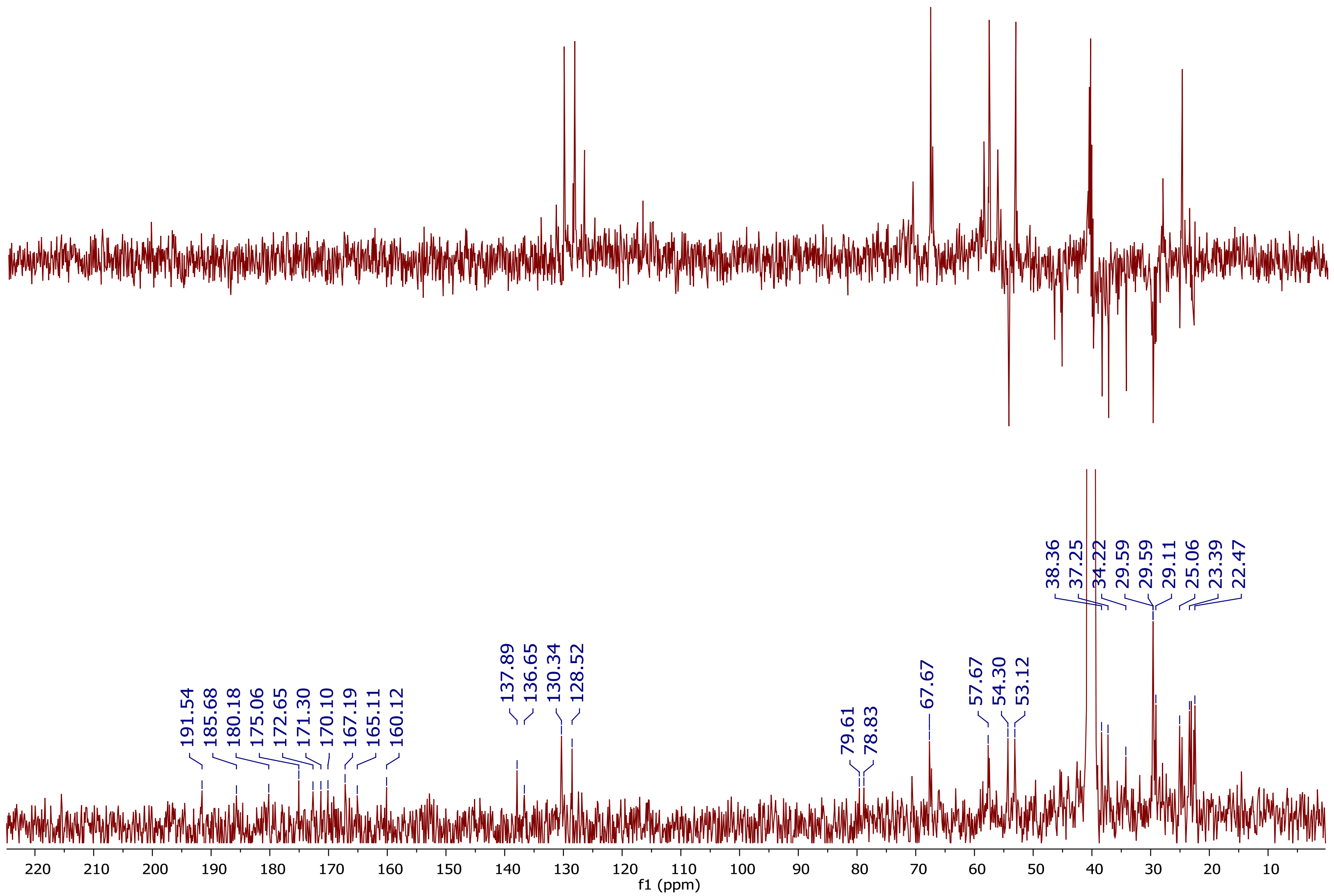

Figure S2. UV, mass spectral data and MS/MS fragmentation data for the most intense chromatographic peaks of the crude ethyl acetate extract obtained from the ISP2 agar culture of *Actinokineospora* sp. EG49.

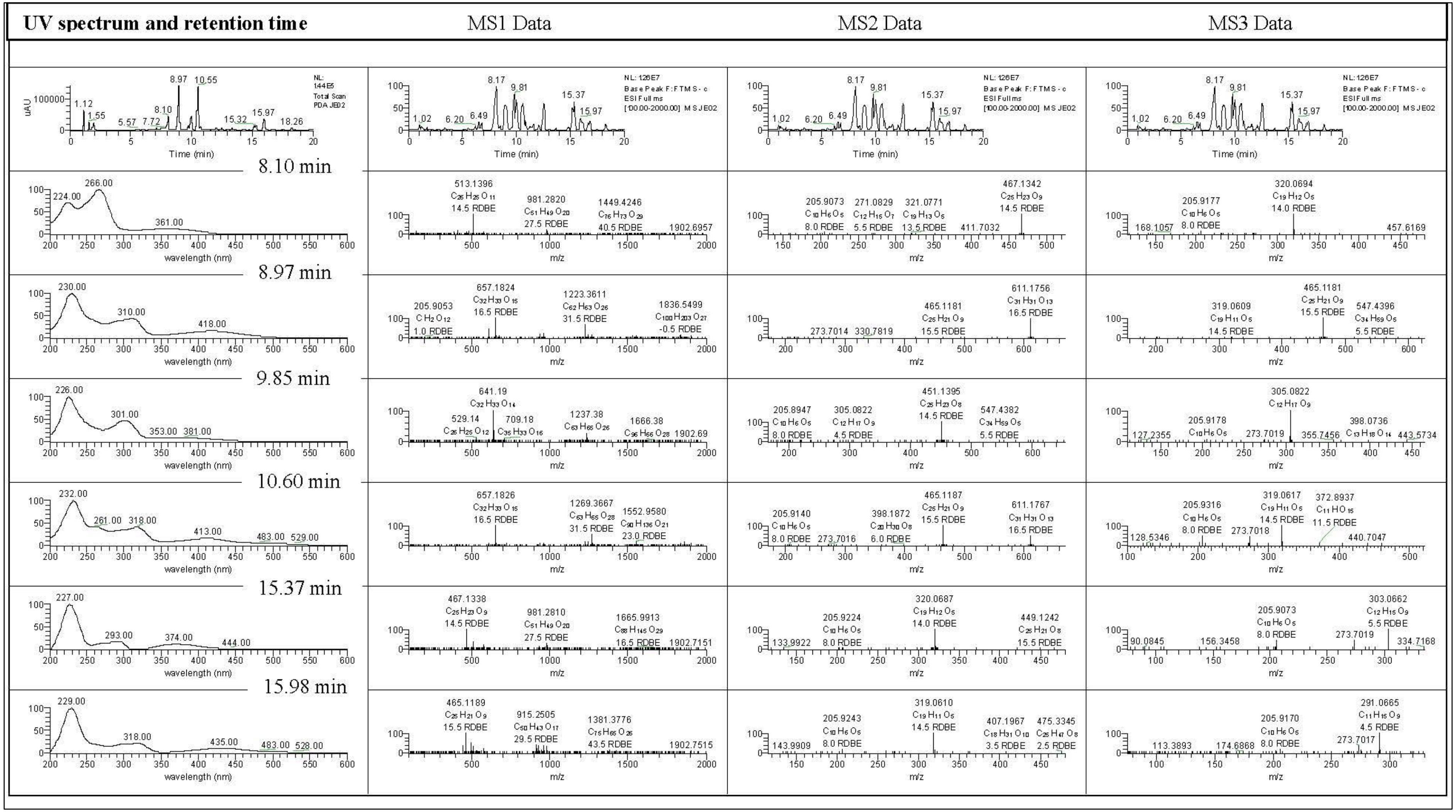

Figure S3. Schematic representation of bioassay-guided fractionation of *Actinokineospora* sp. EG49 extract. ***** Active fractions against *Trypanosoma brucei brucei* strain TC 221 with growth inhibition more than 50%.

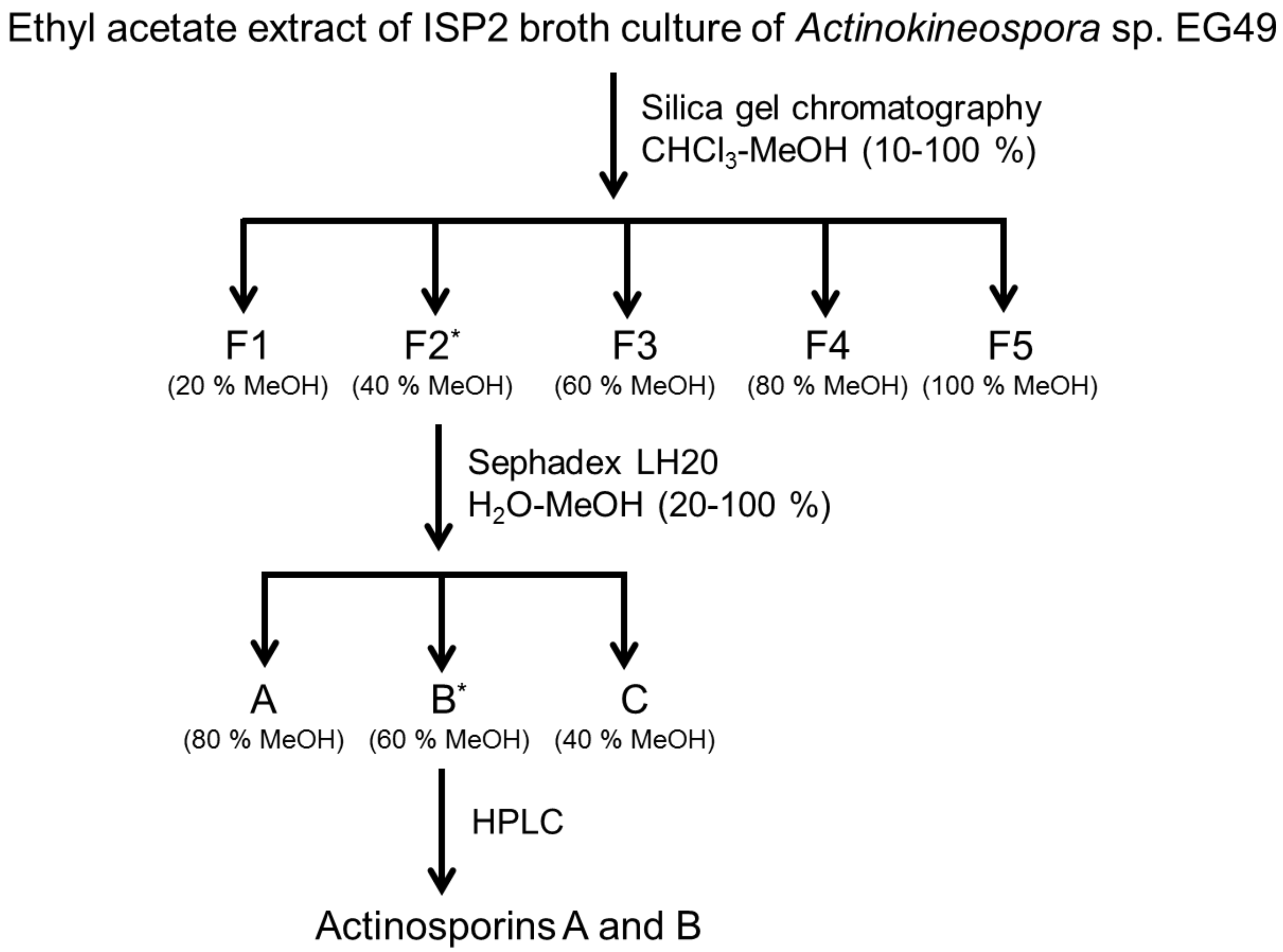

Figure S4. Proton spectrum for Actinosporin A.

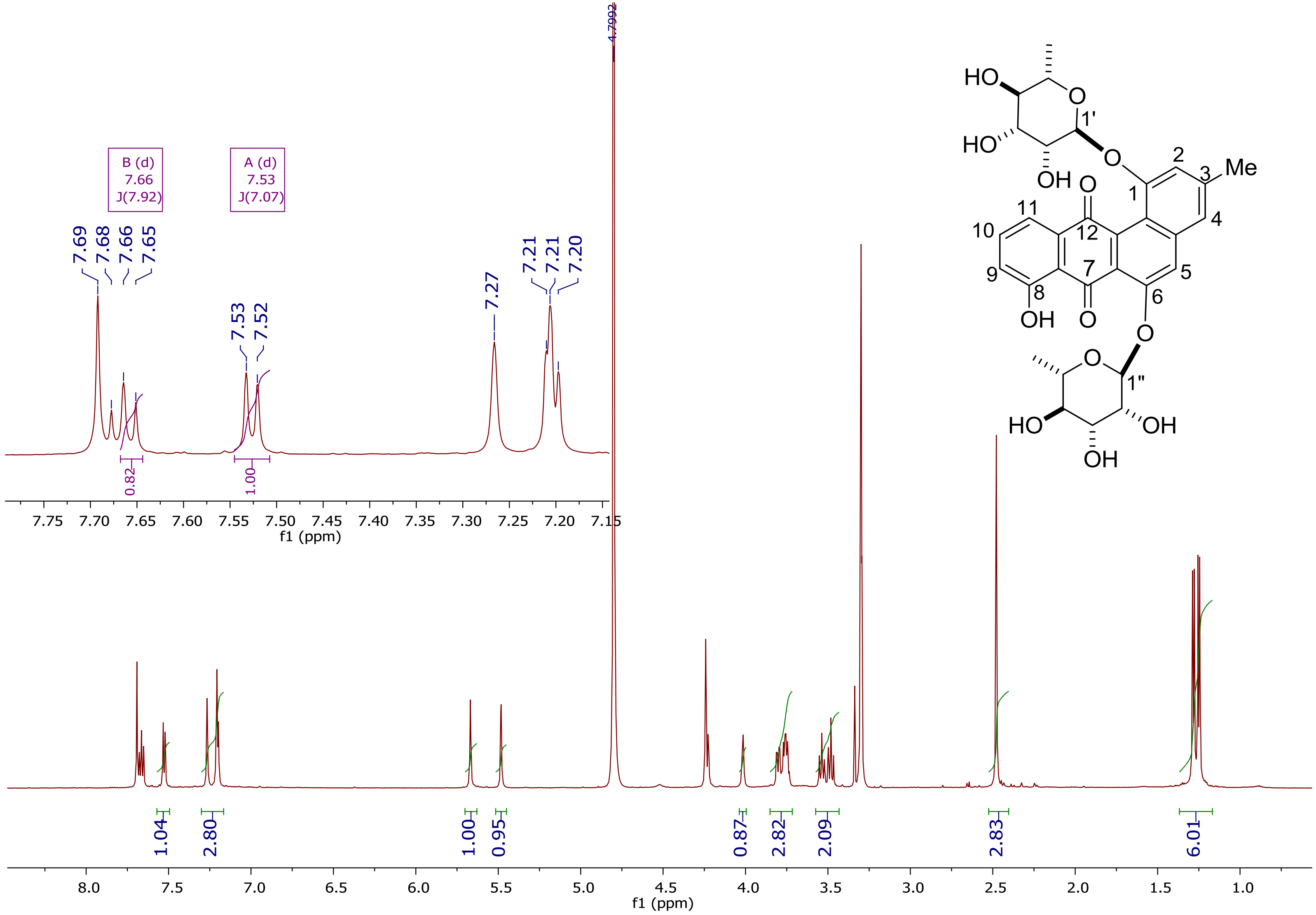

Figure S5. COSY spectrum for Actinosporin A.

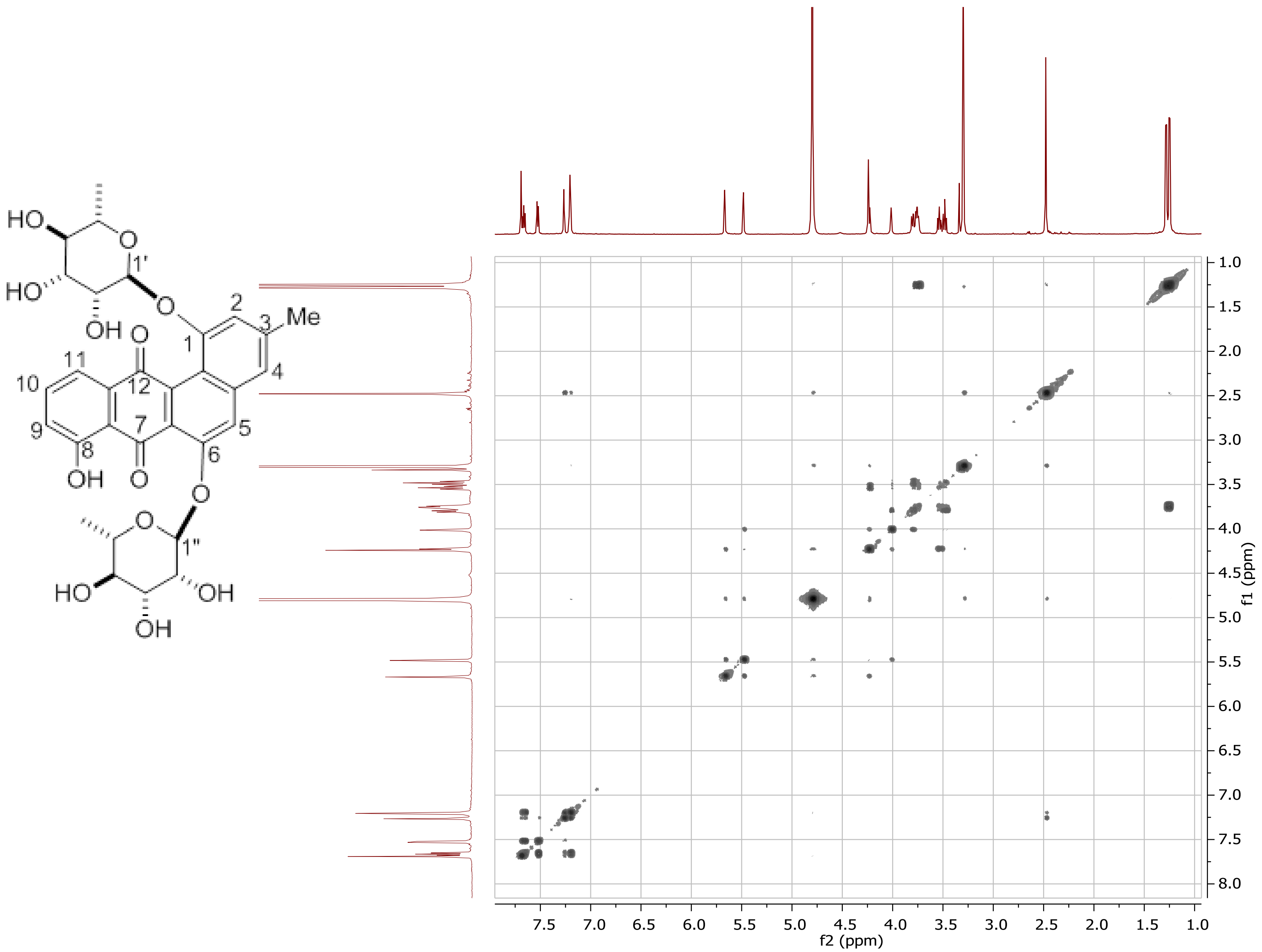

Figure S6. ROESY spectrum for Actinosporin A.

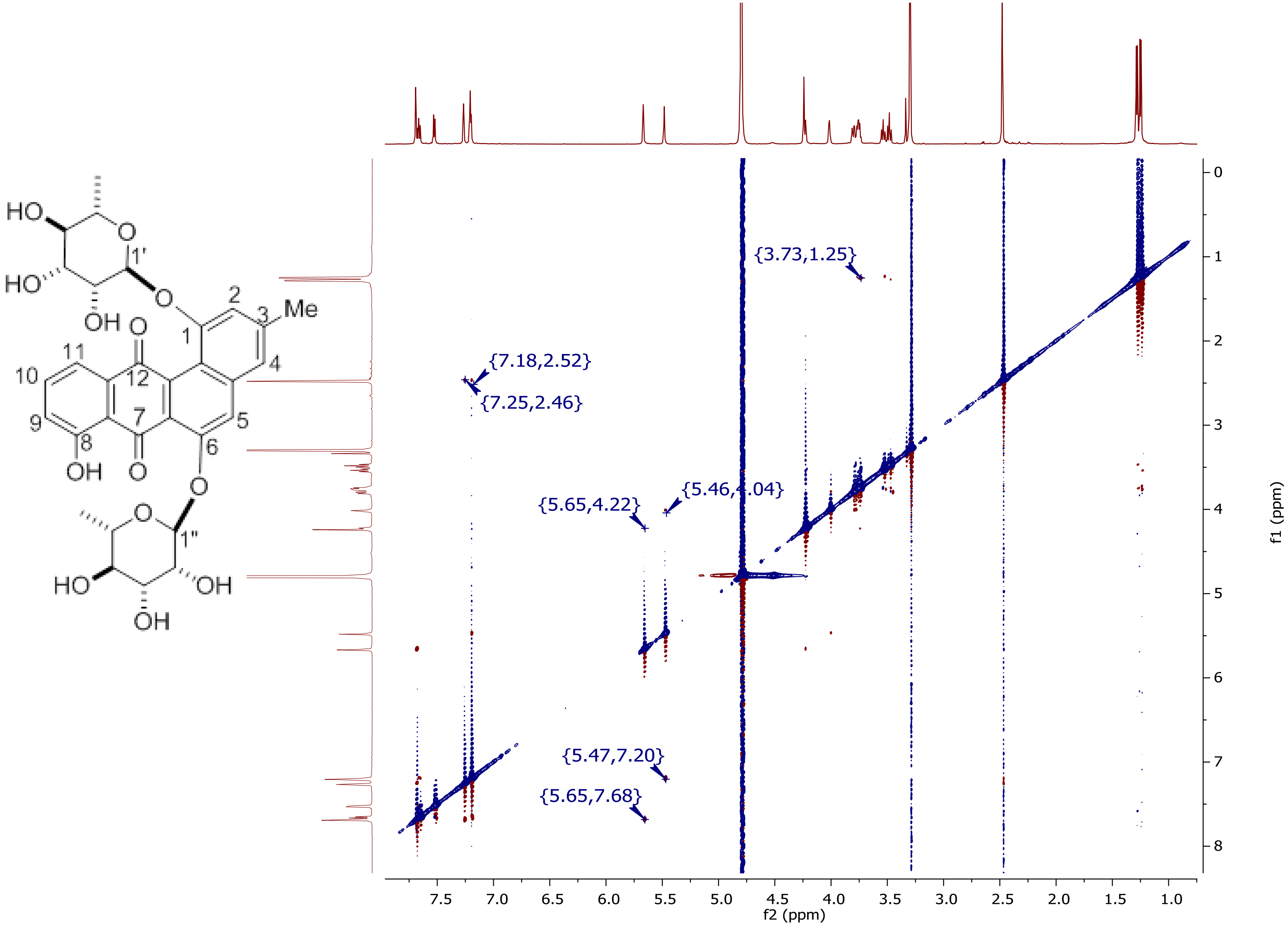

Figure S7. Carbon spectrum for Actinosporin A.

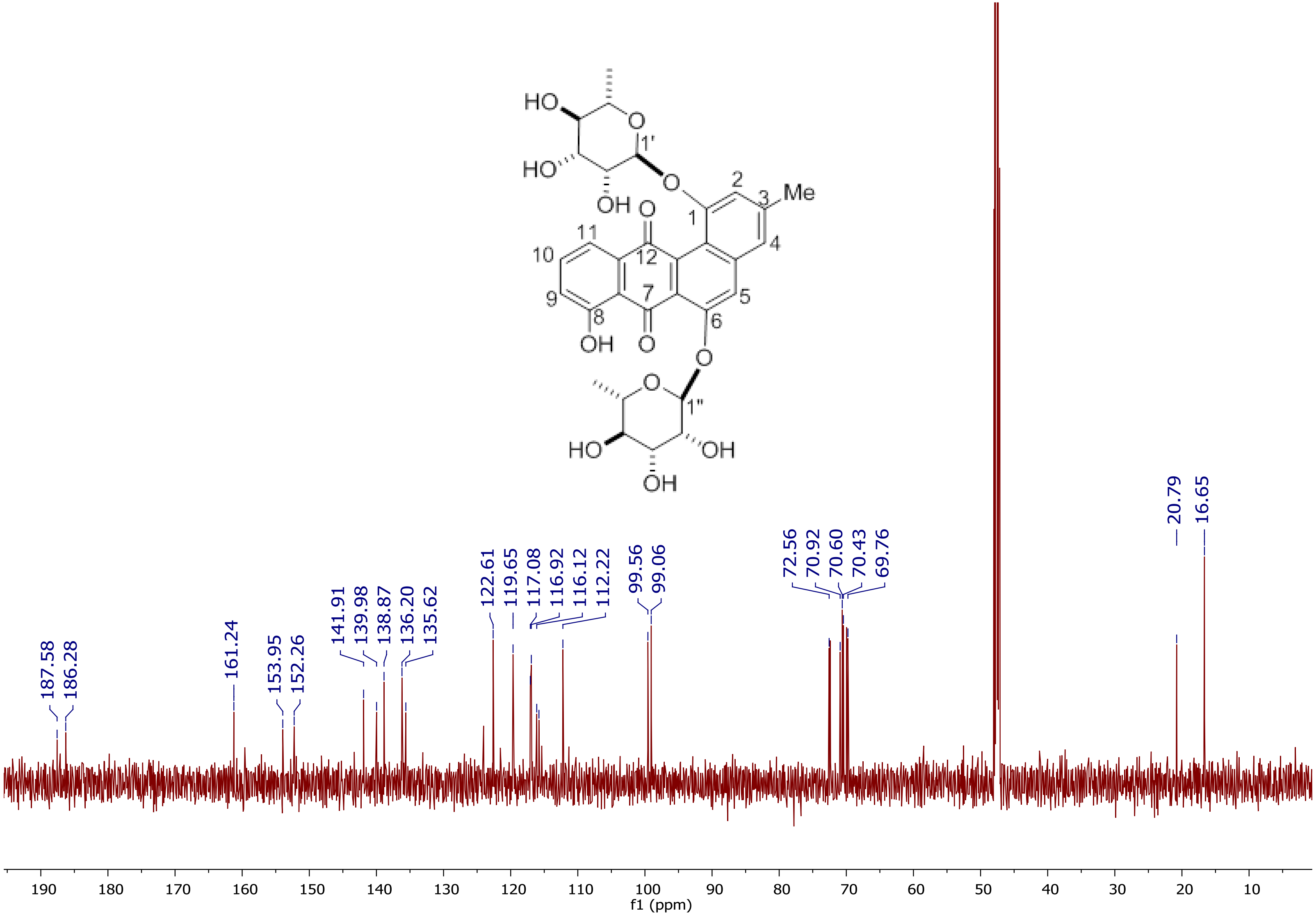

Figure S8. HMBC spectrum for Actinosporin A.

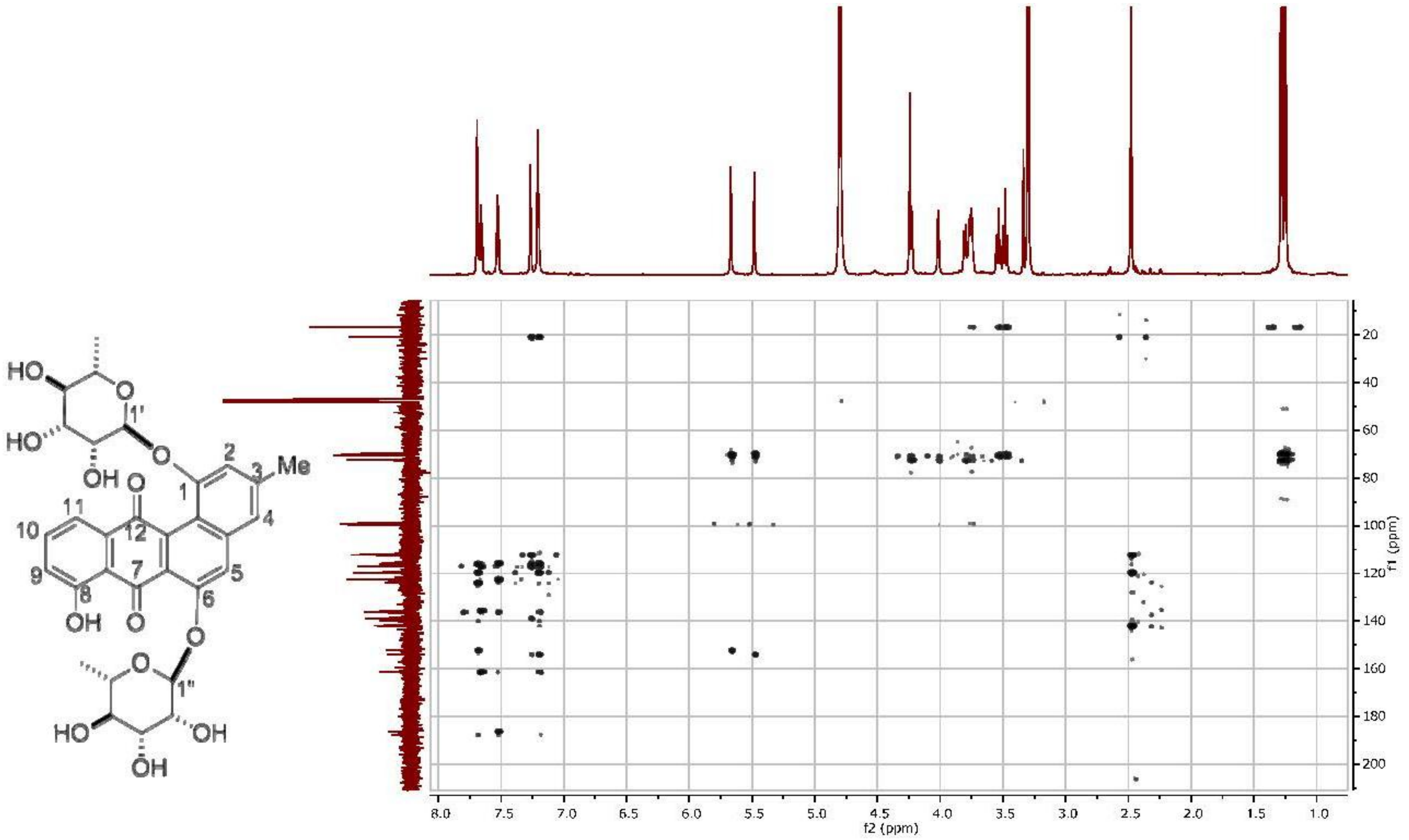

Figure S9. HSQC spectrum for Actinosporin A.

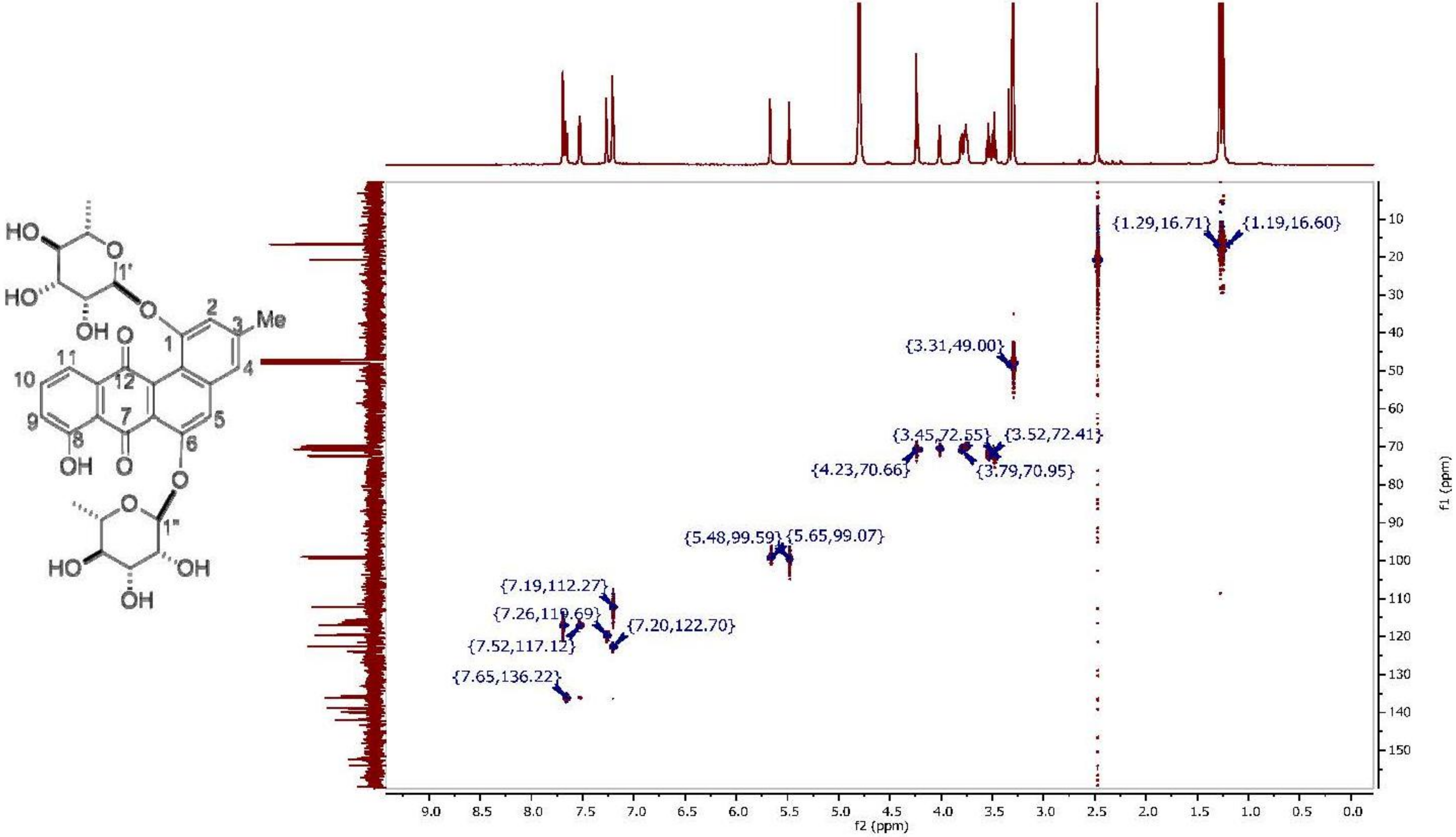

Figure S10. Proton spectrum for Actinosporin B.

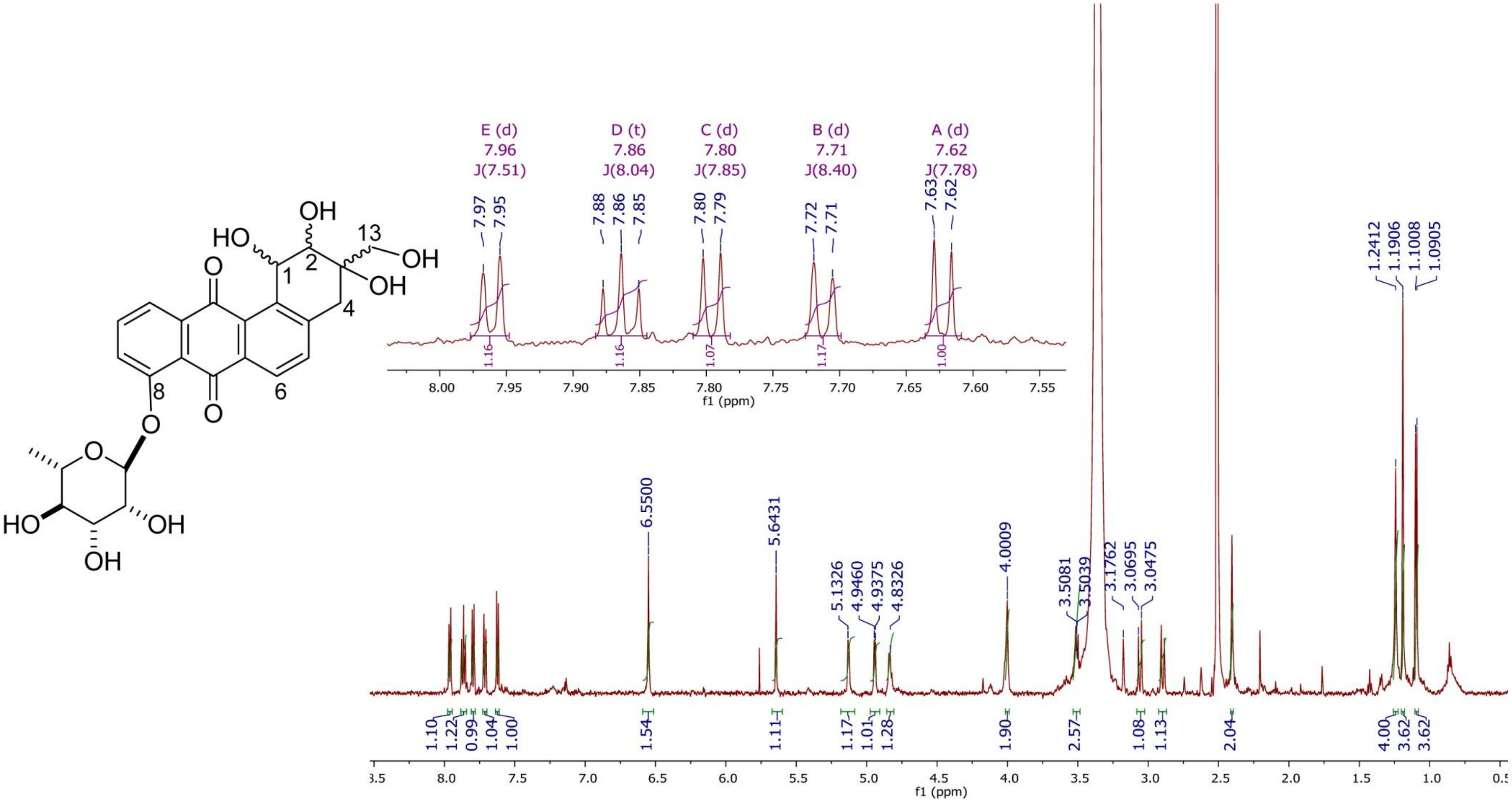

Figure S11. COSY spectrum for Actinosporin A (Full Spectrum).

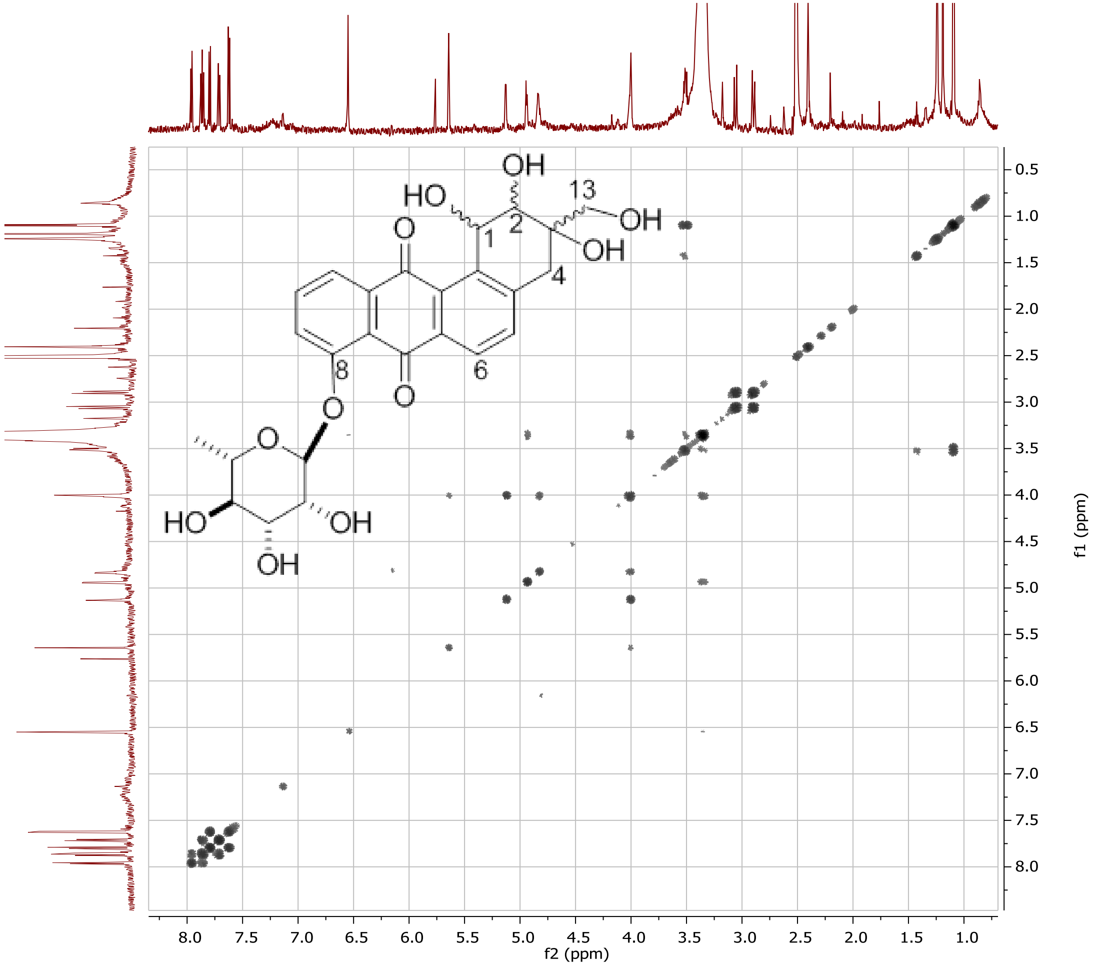

Figure S12. COSY spectrum for Actinosporin B (aromatic region).

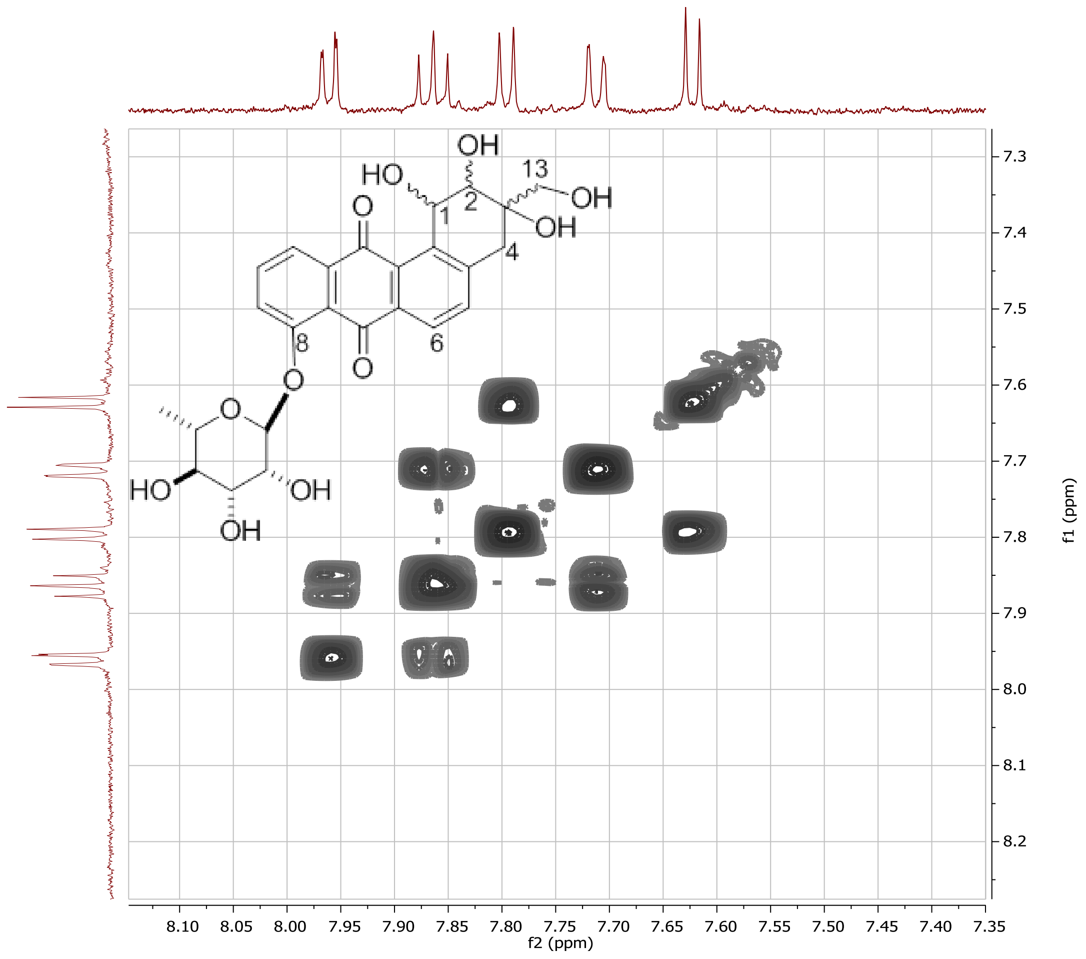

Figure S13. COSY spectrum for Actinosporin B (aliphatic region).

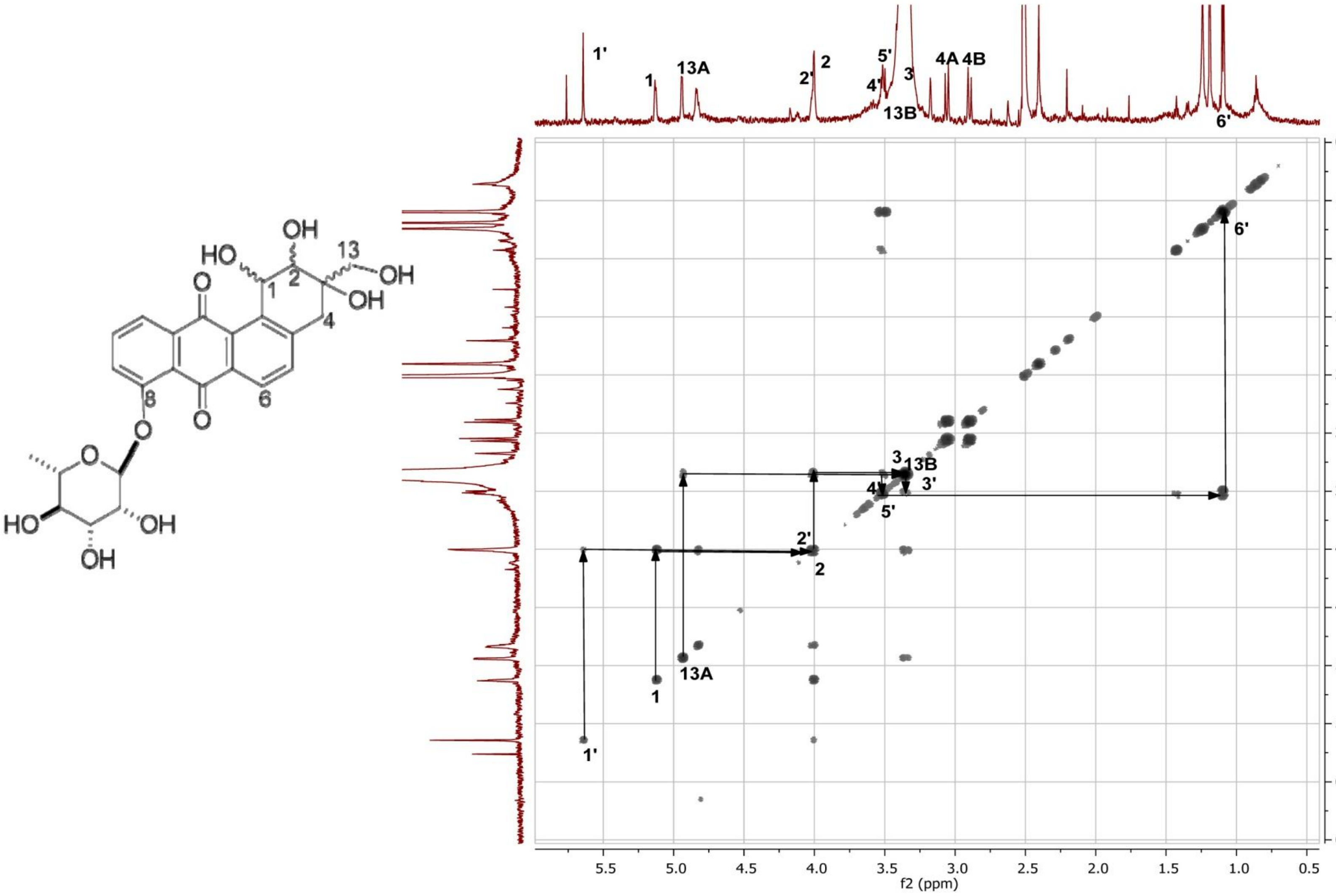

Figure S14. HMBC spectrum for Actinosporin B (aromatic region).

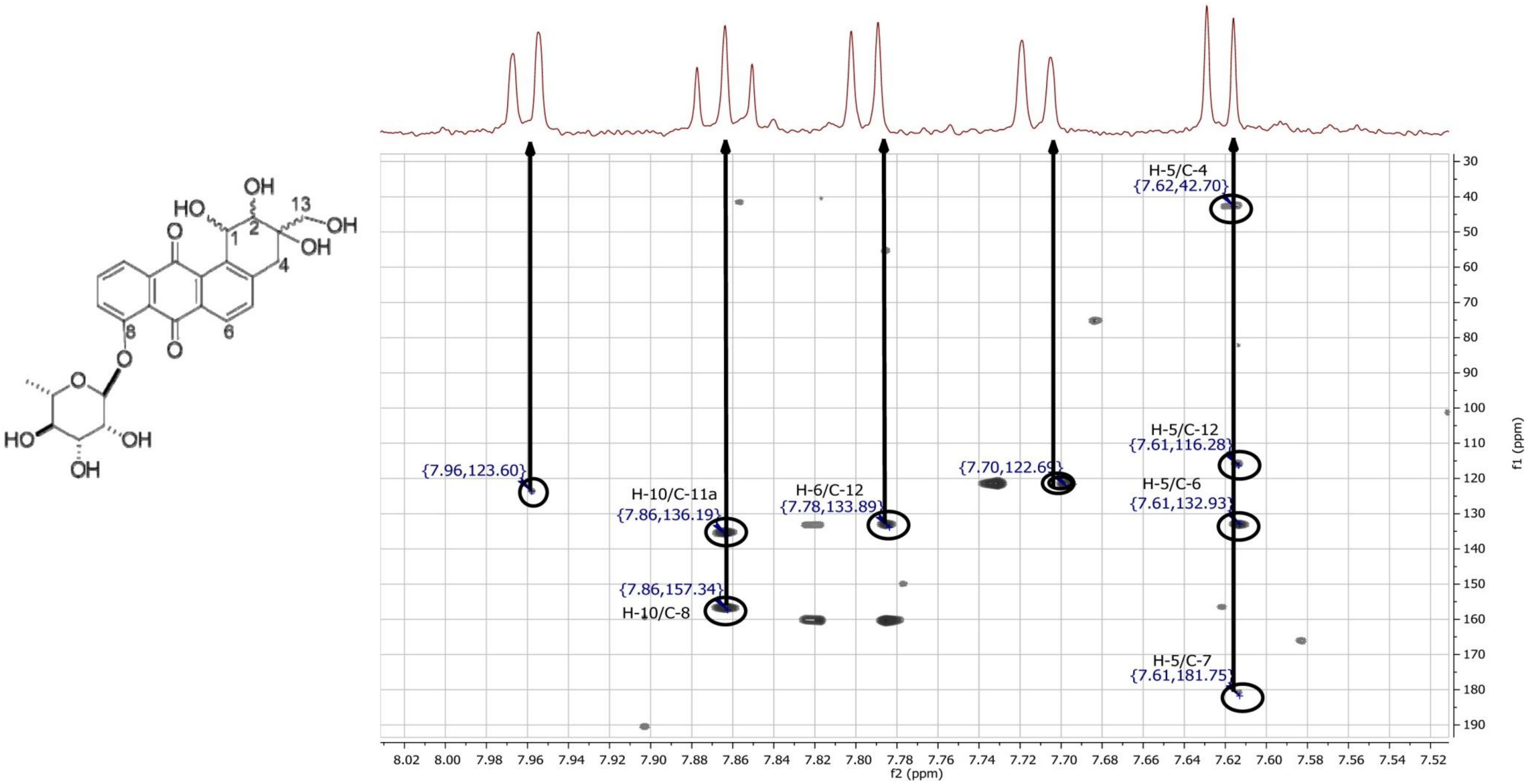

Figure S15. HMBC spectrum for Actinosporin B (aliphatic region).

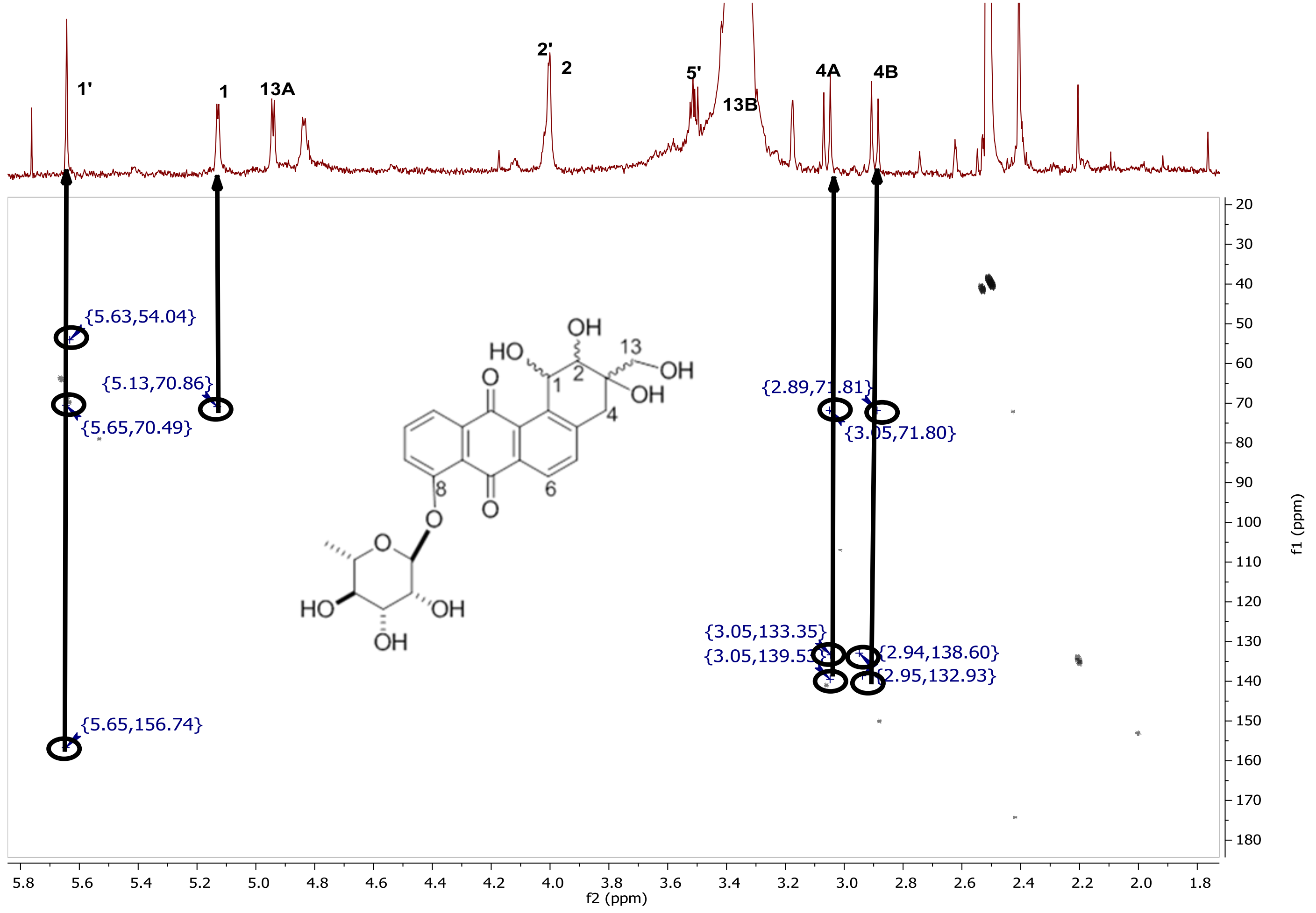

© 2014 by the authors; licensee MDPI, Basel, Switzerland. This article is an open access article distributed under the terms and conditions of the Creative Commons Attribution license (http://creativecommons.org/licenses/by/3.0/).
